# Effect of bridgmanite-ferropericlase grain size evolution on Earth’s average mantle viscosity: implications for mantle convection in early and present-day Earth

**DOI:** 10.1186/s40645-024-00658-3

**Published:** 2024-12-04

**Authors:** Jyotirmoy Paul, Gregor J. Golabek, Antoine B. Rozel, Paul J. Tackley, Tomoo Katsura, Hongzhan Fei

**Affiliations:** 1 Bayerisches Geoinstitut, University of Bayreuth, Universitätsstraße 30, 95447 Bayreuth, Germany; 2 Centre for Planetary Habitability, University of Oslo, 0316 Oslo, Norway; 3 Institute of Geophysics, Department of Earth and Planetary Sciences, ETH Zürich, Sonneggstrasse 5, 8092 Zürich, Switzerland; 4 School of Earth Sciences, Zhejiang University, Hangzhou, China

**Keywords:** Numerical modeling, Grain size, Lower mantle, Viscosity, Bridgmanite-ferropericlase, Grain size-dependent viscosity, Composite rheology

## Abstract

Recent experimental investigations of grain size evolution in bridgmanite-ferropericlase assemblages have suggested very slow growth for these bimodal phases. Despite numerous speculations on grain size-dependent viscosity, a comprehensive test with realistic grain size evolution parameters compatible with the lower mantle has been lacking. In this study, we develop self-consistent 2-D spherical half-annulus geodynamic models of Earth’s evolution using the finite volume code StagYY to assess the role of grain size on lower mantle viscosity. We explore several models with and without grain size evolution to compare their effects on mantle viscosity. In models with grain size evolution, we consider three scenarios: (1) uniform grain growth throughout the entire mantle with a composite rheology, (2) different grain growth in the upper and lower mantle with a composite rheology, and (3) different grain growth in the upper and lower mantle with purely diffusion creep rheology. In the case of different grain size evolution, the upper mantle’s grain size evolution law is controlled by forsterite-enstatite grain growth, while the lower mantle’s grain size evolution law is controlled by bridgmanite-ferropericlase grain growth. Our results suggest that mantle viscosity is primarily controlled by temperature, whereas grain size has a minor effect compared to the effect of temperature. We attribute two primary reasons for this: First, the bridgmanite-ferropericlase growth is very slow in the lower mantle and the grain size variation is too small to significantly alter the mantle viscosity. Secondly, if grains grow too fast, thus the mantle deforms in the dislocation creep regime, making viscosity grain size-independent. To establish the robustness of this finding we vary several other model parameters, such as surface yield strength, phase transition grain size reset, different transitional stresses for creep mechanisms, pressure dependence on grain growth, and different grain damage parameters. For all our models, we consistently find that grain size has a very limited effect on controlling lower mantle viscosity in the present-day Earth. However, large grain size may have affected the lower mantle viscosity in the early Earth as larger grains of single phase bridgmanite could increase the viscosity of the early mantle delaying the onset of global convection.

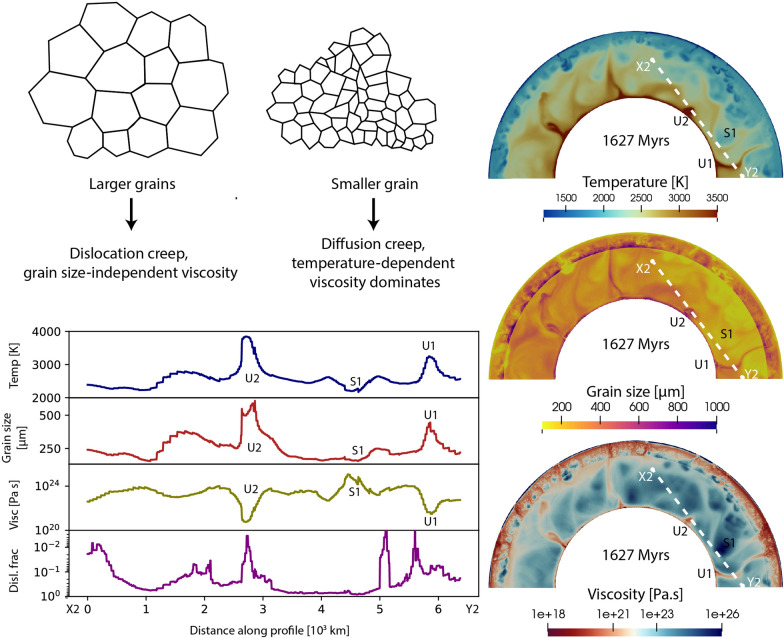

## Introduction

The mantle is one of the primary layers of terrestrial planets and often experiences convective motion. Mantle convection in these bodies plays a crucial role in planetary evolution, influencing key processes such as heat and mass transfer (Li et al. 2018), chemical layering and mixing (Tackley 2000; van Keken et al. 2002), and even geomagnetic reversals (Biggin et al. 2012). Specifically, in the case of Earth, mantle flow directly impacts its habitability by affecting global dynamic topography, sea level changes (Arnould et al. 2018; Conrad and Husson 2009), and interaction with the global climate (Landuyt and Bercovici 2009; Lee et al. 2016; O’Neill and Aulbach 2022). Therefore, understanding the convection patterns in Earth’s mantle is of utmost importance. One of the key intrinsic properties that governs the nature of convection is viscosity, thus the viscosity of Earth-like planetary interiors has been the subject of extensive research spanning several decades. Experimental and numerical studies have revealed that the net viscosity is a result of several deformation mechanisms within Earth’s interior. These mechanisms encompass diffusion creep, dislocation creep, and plasticity (Karato 1984). Notably, dislocation creep is stress-dependent and grain size-independent, whereas diffusion creep is primarily governed by grain size evolution. However, the effect of grain size on mantle viscosity has not been well constrained yet.

A positive correlation between grain size and the viscosity associated with diffusion creep has been shown (Karato 1989); i.e., an increase in grain size results in larger diffusion creep viscosity. Additionally, grain growth is a thermally activated process, with elevated temperatures resulting in larger grain sizes in order to reduce the interfacial energy. Thus, in regions with elevated temperatures, such as mantle plumes, where temperature-dependent viscosity tends to decrease, grain growth can potentially counteract this effect by increasing viscosity (Dannberg et al. 2017; Korenaga 2005). This phenomenon is particularly relevant when diffusion creep dominates the rheological regime. Several previous studies postulated that mantle plumes might exhibit higher viscosities than expected due to the grain size’s influence (Dannberg et al. 2017; Korenaga 2005; Solomatov 1996; Solomatov et al. 2002).

Rudolph et al. (2015) suggested a sudden increase in viscosity in the mid-mantle, while temperature should gradually increase with depth. Fei et al. (2023) interpreted this viscosity increase as an expression of a larger grain growth rate in the deep lower mantle than in the mid-mantle. They argued that the grain growth in the deep lower mantle may be faster than in the mid-mantle due to the possible presence of bridgmanite-enriched ancient mantle structures (BEAMS) in the deep lower mantle (Ballmer et al. 2017).

In addition to grain growth, grain size may also be reduced due to dynamic recrystallization dynamic recrystallization during dislocation creep (Rozel 2012). The high stresses in and around slabs may promote dislocation creep and thus result in dynamic recrystallization. The ensuing reduction in grain size can in turn enhance the diffusion creep. Thus, dynamic recrystallization could effectively produce weaker slabs (Dannberg et al. 2017; Gerya et al. 2021). Extensive shearing within the asthenosphere may also reduce grain size, which could be a potential explanation for the high seismic attenuation zone (low-Q), and low-velocity asthenosphere (Behn et al. 2009; Faul and Jackson 2005; Ramirez et al. 2023). The reduction of grain size in shear zones can further reduce the viscosity (e.g. Mulyukova and Bercovici 2017; Foley 2018), potentially triggering earthquake slip (Thielmann et al. 2015; Thielmann 2018). Additionally, mineral phase changes along the transition zone can reduce grain size (Solomatov and Reese 2008), although it remains challenging to experimentally or observationally constrain grain size in the transition zone. Apart from influencing local-scale mantle viscosity and dynamics, grain size evolution within the planet may influence the planet’s convective regime (Rozel 2012; Foley et al. 2012; Bercovici and Ricard 2014) and the onset of convective instability (Hall and Parmentier 2003).

Despite well-established mathematical models (Rozel et al. 2011; Rozel 2012), the impact of grain size evolution on controlling large-scale lower mantle dynamics has remained speculative. The challenges in understanding the effects of grain size evolution on lower mantle dynamics are twofold. The primary challenge lies in experimental limitations, given the difficulty in obtaining samples from the deep Earth, which makes it difficult to determine the actual grain size in mantle rocks. Therefore, the grain size distribution in the mantle should be inferred by assuming the conditions and history experienced by the mantle rocks using the grain growth rates determined by laboratory experiments (cf. Fei et al. 2021; Yamazaki et al. 1996). For this reason, some experimental studies have estimated grain growth in specific mineral systems such as calcite-quartz (Austin and Evans 2007), majorite and stishovite (Yamazaki et al. 2010), forsterite-enstatite (Hiraga et al. 2010), ringwoodite (Yamazaki et al. 2005) and bridgmanite (Fei et al. 2021; Yamazaki et al. 1996). In particular, Fei et al. (2021) determined the grain growth rate of bridgmanite coexisting with ferropericlase at 27 GPa, which approximates the pressure at the top of Earth’s lower mantle. However, the precise law governing grain size evolution remains elusive, particularly in the case of grain size reduction parameters. The second challenge is the implementation of grain size evolution in numerical models, when a composite rheology is included. Although it is typically assumed that the lower mantle deforms mostly in the diffusion creep regime (Karato et al. 1995), evidence suggests that dislocation creep may be dominant in the regions around subducting slabs (Ferreira et al. 2019; McNamara et al. 2002). Theoretical models of Boioli et al. (2017) and Reali et al. (2019) predict widespread dislocation creep in the lower mantle. Hence, for realistic numerical models, we need to consider composite rheology in the lower mantle.

While it has been hypothesized that grain size evolution may play a crucial role in mantle dynamics, this hypothesis has yet to be rigorously tested numerically, with realistic grain growth parameters and composite rheology. Although there is no experimental constraint on grain size reduction parameters, some insights have been obtained for bridgmanite-ferropericlase grain growth in the lower mantle (Fei et al. 2021). Previous data on forsterite-enstatite grain growth (Hiraga et al. 2010) estimate parameters for the upper mantle. In the current study, we develop 2-D spherical half-annulus numerical models of Earth’s evolution using the most up-to-date grain size evolution parameter data set (Fei et al. 2021). These models incorporate a composite rheology and aim to investigate the role of grain size evolution in controlling the rheology and dynamics of the lower mantle. Our primary focus is to understand the evolution of the lower mantle, where diffusion creep is assumed to be the dominant mechanism (Karato et al. 1995; McNamara et al. 2002), making the effect of grain size most significant. Unlike previous studies with uniform grain growth rates throughout the whole mantle, we use distinct grain size evolution laws for the upper and lower mantle, respectively. We compare our results with different growth parameters in the lower mantle and quantitatively assess their impact on mantle rheology and creep mechanisms, ultimately shedding light onto lower mantle dynamics.

## Methods

### Composite mantle rheology

The mantle composite rheology combines dislocation and diffusion creep (Eq. 1), where diffusion creep and dislocation creep are defined in Eqs. (2, 3) (Rozel 2012).

1 $$\begin{aligned} & {\frac{1}{\eta _{\text{creep}}} = \frac{1}{\eta _{\text{df}}} + \frac{1}{\eta _{\text{dis}} }} \end{aligned}$$

2 $$\begin{aligned} & {\eta _{\text{df}} = \eta _{0} \text{exp} \left( \frac{\text{E}_{\text{df}} + \text{PV}_{\text{df}}(\text{P})}{\text{RT}} - \frac{\text{E}_{\text{df}}}{\text{RT}_0}\right) \left( \frac{\mathcal {R}}{\mathcal {R}_\text{r}}\right) ^{\text{m}} } \end{aligned}$$

3 $$\eta _{{{\text{dis}}}} = \eta _{0} {\text{exp}}\left( {\frac{{{\text{E}}_{{{\text{dis}}}} + {\text{PV}}_{{{\text{dis}}}} ({\text{P}})}}{{{\text{RT}}}} - \frac{{{\text{E}}_{{{\text{dis}}}} }}{{{\text{RT}}_{0} }}} \right)\left( {\frac{\tau }{{\tau _{0} }}} \right)^{{1 - {\text{n}}}}$$

$\eta _{{{\text{creep}}}}$ is the effective viscosity, $\eta _{\text{df}}$ and $\eta _{\text{dis}}$ are the diffusion and dislocation creep viscosity, respectively. $\text{E}_{\text{i}}$ and $\text{V}_{\text{i}}\mathrm {(P)}$ are the activation energy and pressure-dependent activation volume, where $\text{i}$ denotes diffusion (df) or dislocation creep (dis), respectively (see Table 1). ${\eta _{0}}$, $\text{T}_{\text{0}}$, $\text{P}$, $\text{R}$ and $\text{T}$ are the reference viscosity, reference temperature, pressure, universal gas constant and temperature (Table 1). ${\tau }$ is the second invariant of shear stress, ${\tau _0}$ is a reference stress, $\mathcal {R}$ is the average grain size, $\mathcal {R}_r$ is the reference grain size set to 100 μm, while $\text{m}$ and $\text{n}$ are the diffusion and dislocation creep exponent, respectively (see Table 1). To incorporate a more realistic activation enthalpy, we adjust the activation volume with pressure using the fit of Tackley et al. (2013) to the ab initio results of Ammann et al. (2009) for bridgmanite and Ammann et al. (2010) for post-perovskite,

4 $${\text{V}}_{{\text{i}}} \left( {\text{P}} \right) = {\text{V}}_{{\text{i}}}^{0} {\text{exp}}\left( { - \frac{{\text{P}}}{{{\text{P}}_{{{\text{decay}}}} }}} \right)$$

Values of $\text{P}_{\text{decay}}$ for different components and creep regimes are given in Table 1. The maximum brittle yield strength ${\tau _{\text{y,brittle}}}$ is given by Byerlee’s law, $\tau _{{{\text{y}},{\text{brittle}}}} = {\text{c}}_{{\text{f}}} {\text{P}}$, where ${\text{c}}_{{\text{f}}}$ is the friction coefficient. The ductile yield strength $\tau _{\text{y, ductile}}$ linearly increases with pressure considering the surface yield stress $\tau _{\text{y,surf}}$, which is given by the Mohr-Coulomb friction criterion, ${\tau}_{\text{y,ductile}} = {c_{{{\tau}_y}}} P + {\tau}_{y,surf}$, where ${\text{c}}_{{\tau _{{\text{y}}} }}$ is the yield stress gradient. The plastic viscosity ($\eta _{{{\text{pl}}}}$) is determined as,

5 $${\eta}_{{\text{pl}}} = \frac{ {\tau}_{{\text{y}}} }{{2\dot{\epsilon }}}$$

$\tau _{{\text{y}}} = {\text{min}}\left( {\tau _{{{\text{y}},{\text{ductile}}}} ,\tau _{{{\text{y}},{\text{brittle}}}} } \right)$ and ${\dot{\epsilon }}$ is the second invariant of the strain-rate tensor. The net viscosity is given as,

6 $$\begin{aligned} \eta = \text{min} (\eta _{\text{creep}}, \eta _{\text{pl}}) \end{aligned}$$

**Table 1:** List of model parameters

	Value
*Model parameters*	
Surface temperature	300 K
Initial core-mantle boundary temperature	4800 K
Surface thermal expansivity	3.0 $$\times$$$$10^{-5}$$$$\hbox {K}^{-1}$$
Surface yield strength ($\tau _{\text{y,surf}})$	20/30/40 MPa
Ductile yield strength gradient ($\text{c}_{\tau _{\text{y}}})$	0.001
Friction coefficient ($\text{c}_{\text{f}}$)	0.6
Reference viscosity (${\eta _{0}}$)	$10^{21}$ Pa.s
Post-perovskite viscosity drop	0.1
Reference temperature ($\mathrm {T_0}$)	1800 K
Reference stress (${\tau _0}$)	$10^6$/$10^8$ Pa
*Mineral system parameters*	(cf. Gülcher et al. 2022; Schierjott et al. 2020)
Olivine system	
Surface density	3240 kg $\hbox {m}^{-3}$
Phase transition depths	410/660/2740 km
Phase transition temperature	1600/1900/2300 K
Density changes at phase transitions	180/435/61.6 kg $\hbox {m}^{-3}$
Clapeyron slope at phase transitions	2.5/-2.5/10 MPa $\hbox {K}^{-1}$ (Ammann et al. 2010; Karato 1984)
	*Diffusion creep parameters in each phase interval*
Activation energy ($\text{E}_{\text{df}}$)	300/300/370/162 kJ $\hbox {mol}^{-1}$
Activation volume $$(\text{V}_{\text{df}}^{0})$$	$$5.0\times 10 ^{-6}$$/$$5.0\times 10^{-6}$$/$$3.6\times 10^{-6}$$/$$1.4\times 10^{-6}$$$$\hbox {m}^3$$/mol
$\text{P}_{\text{decay}}$	$10^{30}$/$10^{30}$/$200\times 10^9$/$1610\times 10^9$ Pa
Grain size exponent (m)	3
	*Dislocation creep parameters in each phase interval* (Ammann et al. 2010; Karato and Wu 1993)
Activation energy ($\text{E}_{\text{dis}}$)	530/530/370/162 kJ $\hbox {mol}^{-1}$
Activation volume ($$\text{V}_{\text{dis}}^{0}$$)	$$14\times 10 ^{-6}$$/$$14\times 10^{-6}$$/$$3.6\times 10^{-6}$$/$$1.4\times 10^{-6}$$$$\hbox {m}^3$$/mol
$\text{P}_{\text{decay}}$	$10^{30}$/$10^{30}$/$200\times 10^9$/$1610\times 10^9$ Pa
Stress exponent (n)	3.5
Pyroxene-garnet system	
Surface density	3080 kg $\hbox {m}^{-3}$
Phase transition depths	40/400/720/2740 km
Phase transition temperature	1000/1600/1900/2300 K
Density changes at phase transitions	350/150/350/61.6 kg $\hbox {m}^{-3}$
Clapeyron slope at phase transitions	0/1/1/10 MPa $\hbox {K}^{-1}$
	*Diffusion creep parameters in each phase interval* (Ammann et al. 2010; Karato and Wu 1993)
Activation energy ($\text{E}_{\text{df}}$)	300/300/300/370/162 kJ $\hbox {mol}^{-1}$
Activation volume ($$\text{V}_{\text{df}}^{0}$$)	$$5.0\times 10^{-6}$$/$$5.0\times 10^{-6}$$/$$5.0\times 10^{-6}$$/$$3.6\times 10 ^{-6}$$/$$1.4\times 10^{-6}$$$$\hbox {m}^3$$/mol
$\text{P}_{\text{decay}}$	$10^{30}$/$10 ^{30}$/$10^{30}$/$200\times 10^9$/$1610\times 10^9$ Pa
Grain size exponent (m)	3
	*Dislocation creep in each phase interval* (Ammann et al. 2010; Karato and Wu 1993)
Activation energy ($\text{E}_{\text{dis}}$)	530/530/530/370/162 kJ $\hbox {mol}^{-1}$
Activation volume ($$\text{V}_{\text{dis}}^{0}$$)	$$14\times 10^{-6}$$/$$14\times 10^{-6}$$/$$14\times 10^{-6}$$/$$3.6\times 10^{-6}$$/$$1.4\times 10^{-6}$$$$\hbox {m}^3$$/mol
$\text{P}_{\text{decay}}$	$10^{30}$/$10 ^{30}$/$10^{30}$/$200\times 10^9$/$1610\times 10^9$ Pa
Stress exponent (n)	3.5
*Grain growth parameters*	
(Fei et al. 2021)	
Pre-exponential factor ($$\text{G}_{0}$$)	$$10^{2.6}$$$$\upmu \text{m}^{\text{p}}/\text{s}$$
Activation energy ($\text{E}_{\text{G}}$)	260 kJ $\hbox {mol}^{-1}$
Grain size exponent ($\text{p}$)	5.2
(Hiraga et al. 2010; Schierjott et al. 2020)	
Pre-exponential factor ($$\text{G}_{0}$$)	$$1.68665 \times 10^9$$$$\upmu \text{m}^{\text{p}}/\text{s}$$
Activation energy ($\text{E}_{\text{G}}$)	400 kJ $\hbox {mol}^{-1}$
Grain size exponent ($\text{p}$)	5
*Grain damage parameters*	
(Schierjott et al. 2020)	
Surface tension	$10^6$ Pa μm
$\lambda _{2}$	3.5966
$\lambda _{3}$	17.81427
$\text{f}_{\text{bot}}$	$10^{-6}/ 10^{-3}$

From Eq. (2) it is evident that grain size can influence the viscosity of the material. As grain size increases due to higher temperatures promoting rapid grain growth, any hot area in the mantle (e.g. mantle plumes) may have a higher viscosity, counteracting the temperature-dependent viscosity (Dannberg et al. 2017; Solomatov 1996; Korenaga 2005). Similarly, small grain sizes in a slab can counteract the effect of temperature-dependent viscosity, thus making slabs less viscous (Dannberg et al. 2017).

### Physics of grain size evolution

Experimental studies (Karato 1989) suggested that grain growth follows a power law relation with time $\text{t}$ as given by Eq. (7).

7 $${\mathcal{R}}^{{\text{p}}} = {\mathcal{R}}_{0}^{{\text{p}}} + {\text{Gt}}$$

$\mathcal {R}_0$ is the initial grain size. For Earth’s mantle material, the value of grain growth exponent $\text{p}$ varies from 2 to 5. Yamazaki et al. (1996) estimated for the growth exponent a higher value of up to 10–11, however this might have been a result of the moisture content in the fine grained powder (cf. Fei et al. 2021.) $\text{G}$ is grain size coarsening factor given by Karato (1989):

8 $$\begin{aligned} \text{G} = \text{G}_0 \text{exp} \left( \frac{-\text{E}_{\text{G}}}{\text{RT}} \right) \end{aligned}$$

$\mathrm {G_0}$ is the pre-exponential factor and $\mathrm {E_G}$ is the activation energy for grain growth. In the early Earth, crystals grow after nucleation in the magma ocean by a process called Ostwald ripening, where crystal growth is controlled by volume diffusion in a magma ocean melt (Solomatov and Reese 2008). In the subsolidus mantle, grain growth is strongly influenced by the presence of a secondary mineral (Solomatov et al. 2002). In the lower mantle, the primary and secondary minerals are bridgmanite and ferropericlase. While pure bridgmanite shows very fast grain growth (Fei et al. 2023), the presence of ferropericlase subdues this growth by Zener pinning (Fei et al. 2021, 2023; Solomatov et al. 2002; Solomatov and Reese 2008). A recent study suggests that the growth rate of bridgmanite can also be influenced by the presence of trivalent cations, such as $\hbox {Fe}^{3+}$ or $\hbox {Al}^{3+}$ (Fei et al. 2024). During solid mantle convection, the creep of bridgmanite grains produces dislocations within the grains causing the subsequent grain size reduction due to dynamic recrystallization (Solomatov and Reese 2008; Ricard and Bercovici 2009). Thus, grain growth kinetics is a continuous process that is controlled by grain growth and grain damage until they reach an equilibrium grain size. Apart from dynamic recrystallization, grain size is also expected to be affected by phase transitions, which can reduce the grain size significantly (Solomatov 1996; Solomatov and Reese 2008). Combining all physical processes of grain growth and reduction, Rozel et al. (2011) developed a mathematical description of rate of change of grain size (Eq. 9).

9 $$\frac{{{\text{d}}{\mathcal{R}}}}{{{\text{dt}}}} = \frac{{\text{G}}}{{{\text{p}}{\mathcal{R}}^{{{\text{p}} - 1}} }} - {\text{f}}_{{\text{G}}} \frac{{{\mathcal{R}}^{2} }}{{3\gamma }}\frac{{\lambda _{3} }}{{\lambda _{2} }}\tau _{{\text{d}}} :\dot{\epsilon }_{{{\text{dis}}}}$$

The first term on the right hand side of Eq. (9) is the grain growth rate, which is the time derivative of Eq. (7). The second term on the right hand side of Eq. (9) calculates grain size reduction. $\mathrm {f_G}$ is a dimensionless function of temperature that calculates the fraction of work that goes into creating new grain boundaries. A simplified form of $\mathrm {f_G}$ is given by Schierjott et al. (2020)

10 $$\begin{aligned} \text{f}_{\text{G}} = \text{f}_{\text{top}} \left( \frac{\text{f}_{\text{bot}}}{\text{f}_{\text{top}}}\right) ^{\frac{\text{T}-300}{\text{T}_{\text{CMB}}-300}} \end{aligned}$$

$\mathrm {T_{CMB}}$ is the core-mantle boundary (CMB) temperature and $\mathrm {f_{bot}}$ and $\mathrm {f_{top}}$ are the minimum and maximum grain damage at minimum and maximum temperature, respectively. Previous calculations suggested that the value of $\mathrm {f_G}$ should vary between 1 and $10^{-10}$ (Mulyukova and Bercovici 2018; Rozel 2010; Rozel et al. 2011; Schierjott et al. 2020). A higher value of $\mathrm {f_G}$ favours more dynamic recrystallization, while increasing the value of $\mathrm {f_{bot}}$ ensures more grain damage. In our models we keep $\mathrm {f_{top}}$ at $10^{-1}$ and $\mathrm {f_{bot}}$ at $10^{-6}$, except in one model (M16), where $\mathrm {f_{bot}}$ is kept at $10^{-3}$.

Furthermore ${\gamma }$ is the surface tension, ${\frac{\lambda _3}{\lambda _2}}$ is a dimensionless term related to the grain size distribution (see Table 2) and $\tau _{{\text{d}}} :\dot{\epsilon }_{{{\text{dis}}}}$ is shear heating due to dislocation creep. When the growth and damage rates are balanced, i.e., ${\frac{\text{d}\mathcal {R}}{\text{dt}} = 0}$, the equilibrium grain size can be calculated as,

11 $$\begin{aligned} {\mathcal {R}_{\text{eq}} = \left( \frac{3\text{G}\gamma \lambda _2}{\text{pf}_{\text{G}} \lambda _3 \tau _{\text{d}} : \dot{\epsilon }_{\text{dis}}}\right) ^{\frac{1}{\text{p}+1}} } \end{aligned}$$

### Numerical model

We develop self-consistent numerical models of 4.5 Gyrs of Earth evolution in a 2-D spherical half-annulus geometry using the finite volume code StagYY (Tackley 2008; Hernlund and Tackley 2008). The code solves the equations for conservation of mass, momentum and energy on a staggered grid where viscosity, pressure and density are defined in the cell centre and velocities are calculated at the cell walls. The code assumes a viscoplastic rheology in a compressible mantle with infinite Prandtl number. The model domain is discretized into ${512 \times 96}$ cells, which translates into a vertical resolution averaging $\sim$30 km but refined to $\sim$ 10 km near the top. Lagrangian tracers track the evolution of temperature, composition and grain size, with an average of 15 per cell. This is a reasonable resolution for resolving lower mantle heterogeneity, as well as being sufficient to understand the overall mantle dynamics. The lower and upper viscosity cut-offs are $10^{16}$ and $10^{26}$ Pa.s, respectively. To make sure that our results are free of biases due to model domain size and resolution, we test two additional models with different geometries, namely full annulus and quarter annulus, employing grid resolutions of 128 $\times$ 64 and 512 $\times$ 64 nodes (see M17 and M18 in Table 2), respectively. Results show that the average mantle viscosity remains the same regardless of the resolution and domain size.

The initial CMB temperature is 4800 K and the core cools down during the mantle evolution (c.f. Tackley 2012; Schierjott et al. 2020). The surface temperature is kept at 300 K with an initial mantle potential temperature of 1800 K. The top and bottom of the model domain employ the free-slip boundary condition, while the sides are periodic. The average (and initial) rock composition corresponds to 80% harzburgite and 20% basalt, which is equivalent to a pyrolitic composition and gives an upper mantle mineralogy of 60% olivine and 40% pyroxene+garnet (Nakagawa et al. 2010; Xu et al. 2008). Olivine undergoes phase transitions at depths of 410, 660, and 2740 km, while pyroxene+garnet experiences phase transitions at depths of 40, 400, 720, and 2740 km. At each phase transition the density jump, reference temperature and Clapeyron slope are specified (see Table 1). A factor 10 viscosity drop is applied at the perovskite to post-perovskite phase transition at 2740 km. Rheological parameters, including activation energy and activation volume, are also modified at all phase intervals. Upper mantle rheological parameters are based on those for olivine from Karato and Wu (1993), while lower mantle rheological parameters are based on fits by Tackley et al. (2013) to ab initio data for bridgmanite Ammann et al. (2009) and post-perovskite Ammann et al. (2010). Rheology calculations adhere to Eqs. (1)–(5), incorporating grain size dependence as described in Eq. (9). All parameters used in these equations are detailed in Table 1. We compare models with composite rheology and with diffusion creep only to study the effect of grain size evolution.

We choose an initial grain size of 100 μm and in most models a reset to 5 μm when any tracer passes through the 410 km, 660 km or post-perovskite phase transitions. In two additional cases we test the phase change grain size reset to 100 μm and 500 μm (see M10 and M11 in Table 2). There is a lack of data on the initial and present-day grain sizes within the mantle. Xenoliths provide some evidence, but these are mostly from the upper mantle (e.g. Skemer and Karato 2008), with even less knowledge about lower mantle grain sizes. We address this issue analytically by rearranging Eq. (7), showing that the initial grain size can be neglected as time progresses,

12 $${\mathcal{R}} = {\text{t}}^{{1/{\text{p}}}} \left( {\frac{{{\mathcal{R}}_{{\text{0}}}^{{\text{p}}} }}{{\text{t}}} + {\text{G}}} \right)^{{1/{\text{p}}}}$$

When $\text{t} \gg \text{1}$, the dependence of the actual grain size on the term associated with the initial grain size $\frac{{{\mathcal{R}}_{{\text{0}}}^{{\text{p}}} }}{{\text{t}}}$ becomes negligible. Hence, the choice of initial grain size should not affect the result in the long-term.

We use different grain size evolution rates in our models to examine the role of grain size evolution. The reference model M0 does not consider grain size evolution. In model M1 an uniform grain growth law is implemented throughout the whole mantle using forsterite-enstatite grain growth parameters (Hiraga et al. 2010). The next model (M2) employs a more realistic heterogeneous grain growth, in which the upper mantle (0–660 km) grain growth is governed by the forsterite-enstatite grain growth law (Hiraga et al. 2010) and the lower mantle (660-CMB) grain growth is controlled by the bridgmanite-ferropericlase grain growth (Fei et al. 2021). All three models use a composite rheology comprising dislocation creep, diffusion creep and plasticity. In model M3 dislocation creep is turned off, but the different grain growth laws in the upper and lower mantle are included. Additional models test the effect of different surface yield stresses (${\tau _{\text{y,surf}}}$, see Table 1) of 20, 30 and 40 MPa, and phase boundary grain size reset values of 5, 100 and 500 μm. We have also tested the effect of transitional stresses, pressure dependence on grain growth and different grain damage parameters. This produces in total 19 models to investigate the role of grain size evolution on Earth’s lower mantle dynamics. All model descriptions are given in Table 2.

**Table 2:** List of models

Model	Grain growth law	$\tau _{\text{y,surf}}$	$\tau _0$	$f_{bot}$	Creep	Phase boundary grain size reset	geometry and resolution
M0	Nil	20 MPa	$10^6$	$10^{-6}$	Composite	Nil	Half annulus, 512 $\times$ 96
M1	Whole mantle: (Hiraga et al. 2010)	20 MPa	$10^6$	$10^{-6}$	Composite	5 μm	Half annulus, 512 $\times$ 96
M2	Upper mantle: (Hiraga et al. 2010), lower mantle: (Fei et al. 2021)	20 MPa	$10^6$	$10^{-6}$	Composite	5 μm	Half annulus, 512 $\times$ 96
M3	Upper mantle: (Hiraga et al. 2010), lower mantle: (Fei et al. 2021)	20 MPa	$10^6$	$10^{-6}$	Diffusion only	5 μm	Half annulus, 512 $\times$ 96
M4	Whole mantle: (Hiraga et al. 2010)	30 MPa	$10^{6}$	$10^{-6}$	Composite	5 μm	Half annulus, 512 $\times$ 96
M5	Upper mantle: (Hiraga et al. 2010), lower mantle: (Fei et al. 2021)	30 MPa	$10^6$	$10^{-6}$	Composite	5 μm	Half annulus, 512 $\times$ 96
M6	Upper mantle: (Hiraga et al. 2010), lower mantle: (Fei et al. 2021)	30 MPa	$10^6$	$10^{-6}$	Diffusion only	5 μm	Half annulus, 512 $\times$ 96
M7	Whole mantle: (Hiraga et al. 2010)	40 MPa	$10^6$	$10^{-6}$	Composite	5 μm	Half annulus, 512 $\times$ 96
M8	Upper mantle: (Hiraga et al. 2010), lower mantle: (Fei et al. 2021)	40 MPa	$10^6$	$10^{-6}$	Composite	5 μm	Half annulus, 512 $\times$ 96
M9	Upper mantle: (Hiraga et al. 2010), lower mantle: (Fei et al. 2021)	40 MPa	$10^6$	$10^{-6}$	Diffusion only	5 μm	Half annulus, 512 $\times$ 96
M10	Upper mantle: (Hiraga et al. 2010), lower mantle: (Fei et al. 2021)	20 MPa	$10^6$	$10^{-6}$	Composite	100 μm	Half annulus, 512 $\times$ 96
M11	Upper mantle: (Hiraga et al. 2010), lower mantle: (Fei et al. 2021)	20 MPa	$10^6$	$10^{-6}$	Composite	500 μm	Half annulus, 512 $\times$ 96
M12	Whole mantle: (Hiraga et al. 2010)	20 MPa	$10^8$	$10^{-6}$	Composite	5 μm	Half annulus, 512 $\times$ 96
M13	Upper mantle: (Hiraga et al. 2010), lower mantle: (Fei et al. 2021)	20 MPa	$10^8$	$10^{-6}$	Composite	5 μm	Half annulus, 512 $\times$ 64
M14	Common diffusion for creep and grain growth including pressure dependence	20 MPa	$10^6$	$10^{-6}$	Composite	5 μm	Half annulus, 512 $\times$ 64
M15	Upper mantle: (Hiraga et al. 2010), lower mantle: (Fei et al. 2021)	20 MPa	$10^6$	$10^{-6}$	Composite	5 μm without PPV grain size reset	Half annulus, 512 $\times$ 64
M16	Upper mantle: (Hiraga et al. 2010), lower mantle: (Fei et al. 2021)	20 MPa	$10^6$	$10^{-3}$	Composite	5 μm	Half annulus, 512 $\times$ 64
M17	Upper mantle: (Hiraga et al. 2010), lower mantle: (Fei et al. 2021)	20 MPa	$10^6$	$10^{-6}$	Composite	5 μm	Quarter annulus, 128 $\times$ 64
M18	Upper mantle: (Hiraga et al. 2010), lower mantle: (Fei et al. 2021)	20 MPa	$10^6$	$10^{-6}$	Composite	5 μm	Full annulus, 512 $\times$ 64

## Results

### Reference model M0: no grain size evolution

**Fig. 1:**
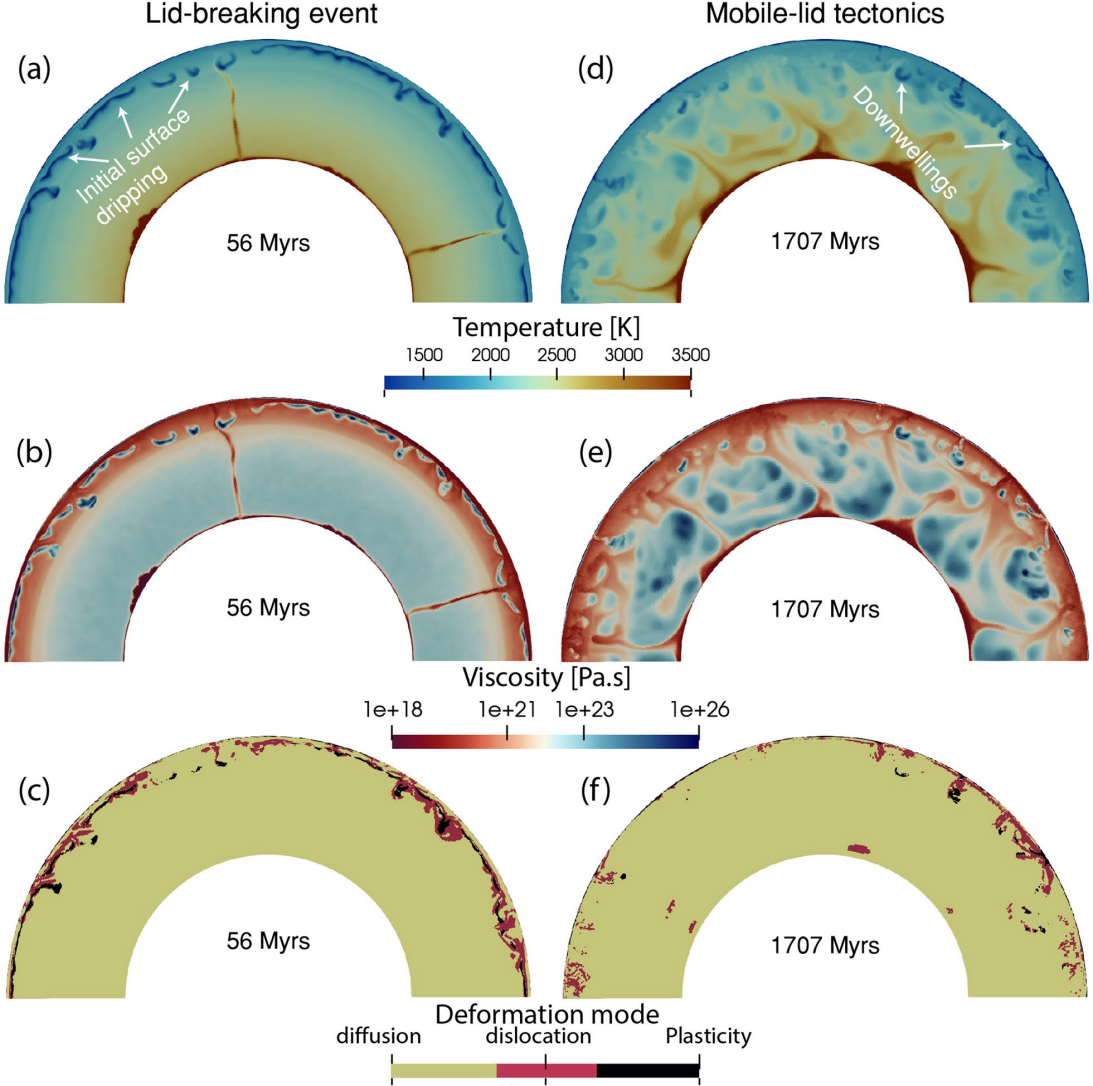
Snapshots of **a**, **d** temperature, **b**, **e** viscosity and **c**, **f** deformation mode from reference model M0. The left panel shows snapshots from the early evolution at 56 Myrs, while the right panel shows snapshots from the late stage featuring a mobile-lid regime at 1707 Myrs

The reference model (M0) has a constant grain size of 100 μm and a surface yield strength of 20 MPa. The lid-breaking event starts within approximately 20 million years (Fig. 1a, b). The initially formed viscous and stagnant lithosphere, gradually transitions into a mobile-lid tectonic regime (supplementary V1, V2). The mantle temperature decreases with time due to convection and by 1.5 Gyrs, the model displays a mobile-lid regime with prominent locations of upwellings and downwellings (Fig. 1d, e, supplementary V1, V2). Diffusion creep remains the dominant creep mechanism in the lower mantle (Fig. 1c, f), while part of the upper mantle is dominated by dislocation creep.

**Fig. 2:**
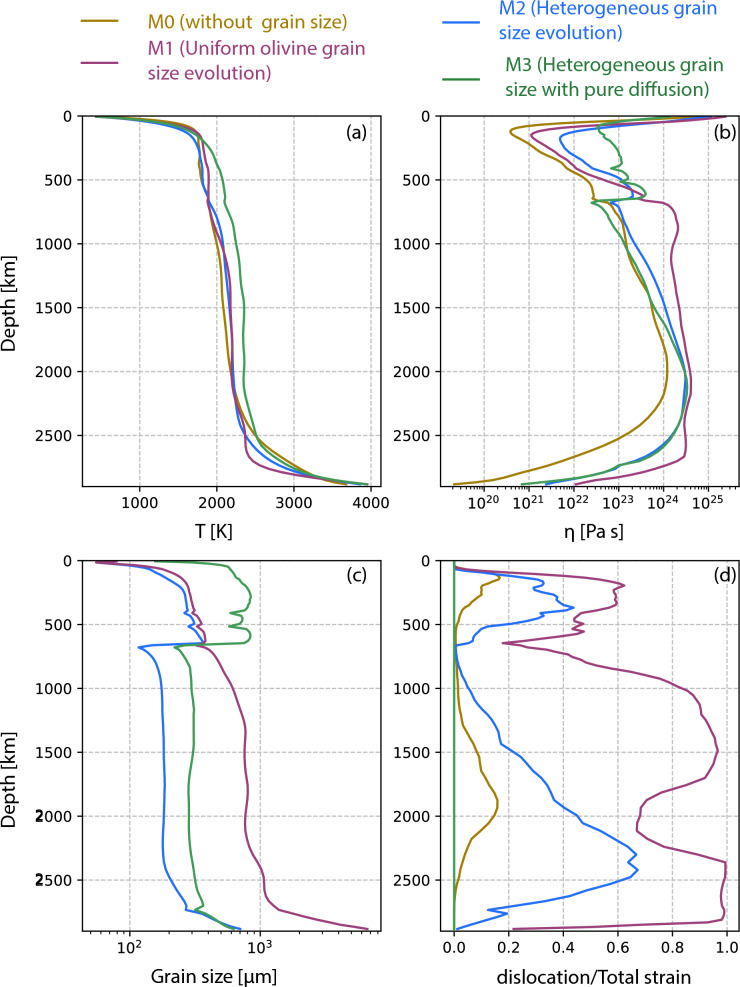
Radial profiles of **a** temperature, **b** viscosity, **c** grain size and **d** deformation mechanism at 3 Gyrs for mobile-lid tectonics. Four lines represent different models described in the index

**Fig. 3:**
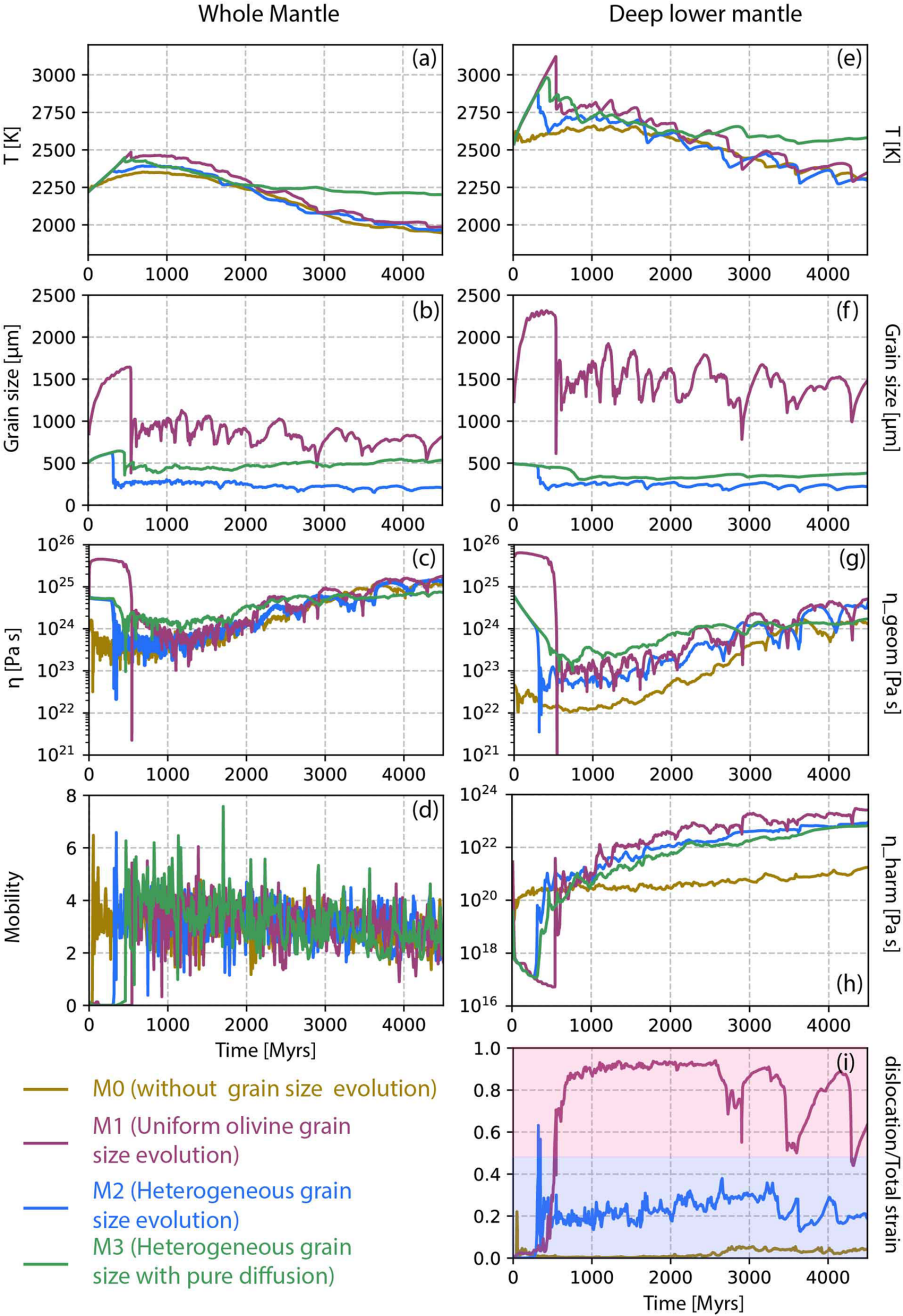
Time evolution of **a**, **e** temperature, **b**, **f** grain size, **c**, **g** geometric mean of viscosity, **d** mobility and **i** deformation mechanism for the whole mantle (left panel) and for the deep lower mantle (right panel). **h** represents the geometric mean and the harmonic mean of the lower mantle viscosity. Different line colors represents models M0 to M3, as given in the index. Shaded regions in **i** indicate the dislocation dominated (light pink) and diffusion dominated (pale blue) regimes

**Fig. 4:**
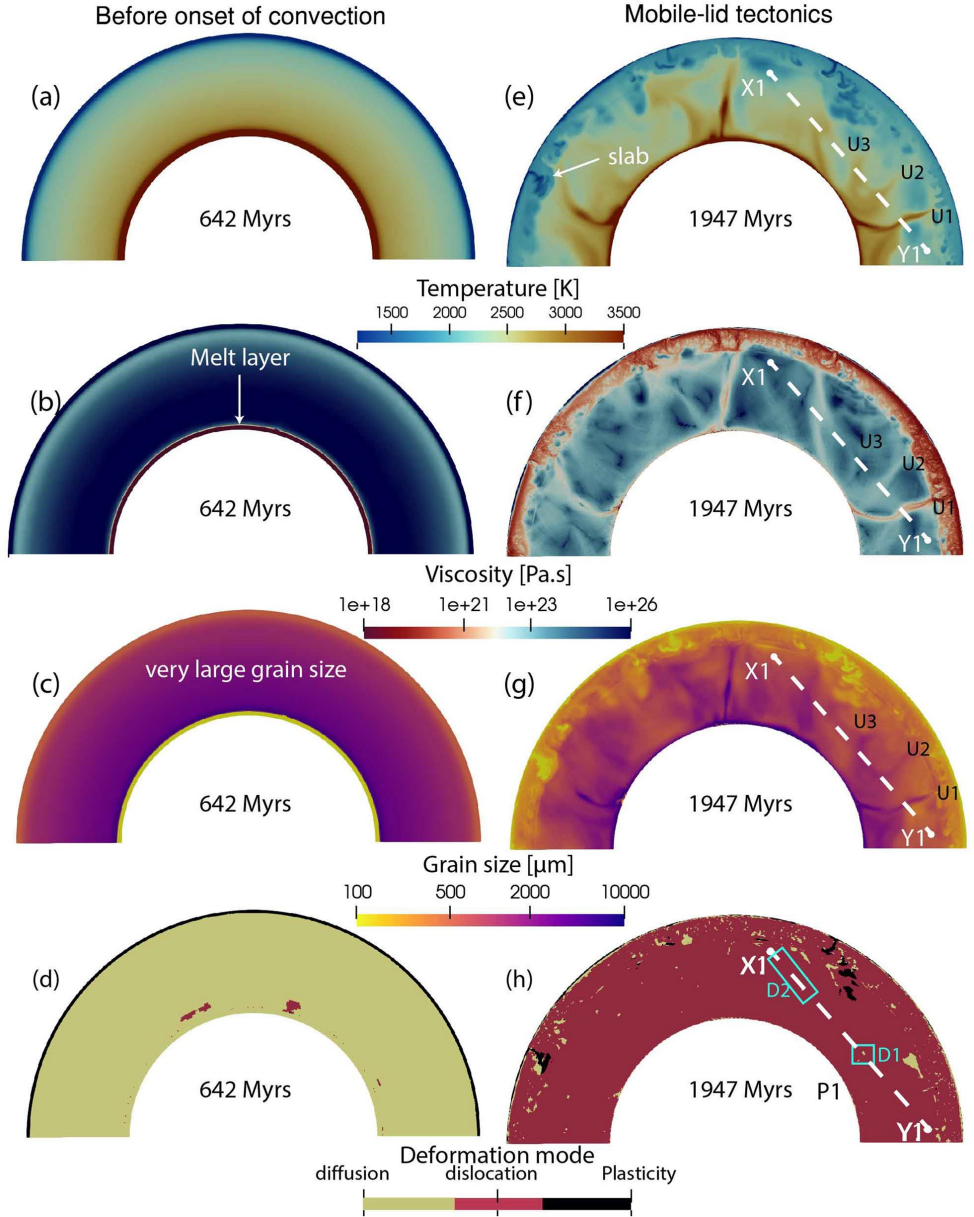
Snapshots of **a**, **e** temperature, **b**, **f** viscosity, **c**, **g** grain size and **d**, **h** deformation mode from model M1. The left panel shows snapshots at 642 Myrs (before the onset of convection) and the right panel shows snapshots at 1947 Myrs, when mobile-lid tectonics is operating. Details on profile X1-Y1, U1, U2, U3, D1 and D2 are discussed in Sect. 4.1

**Fig. 5:**
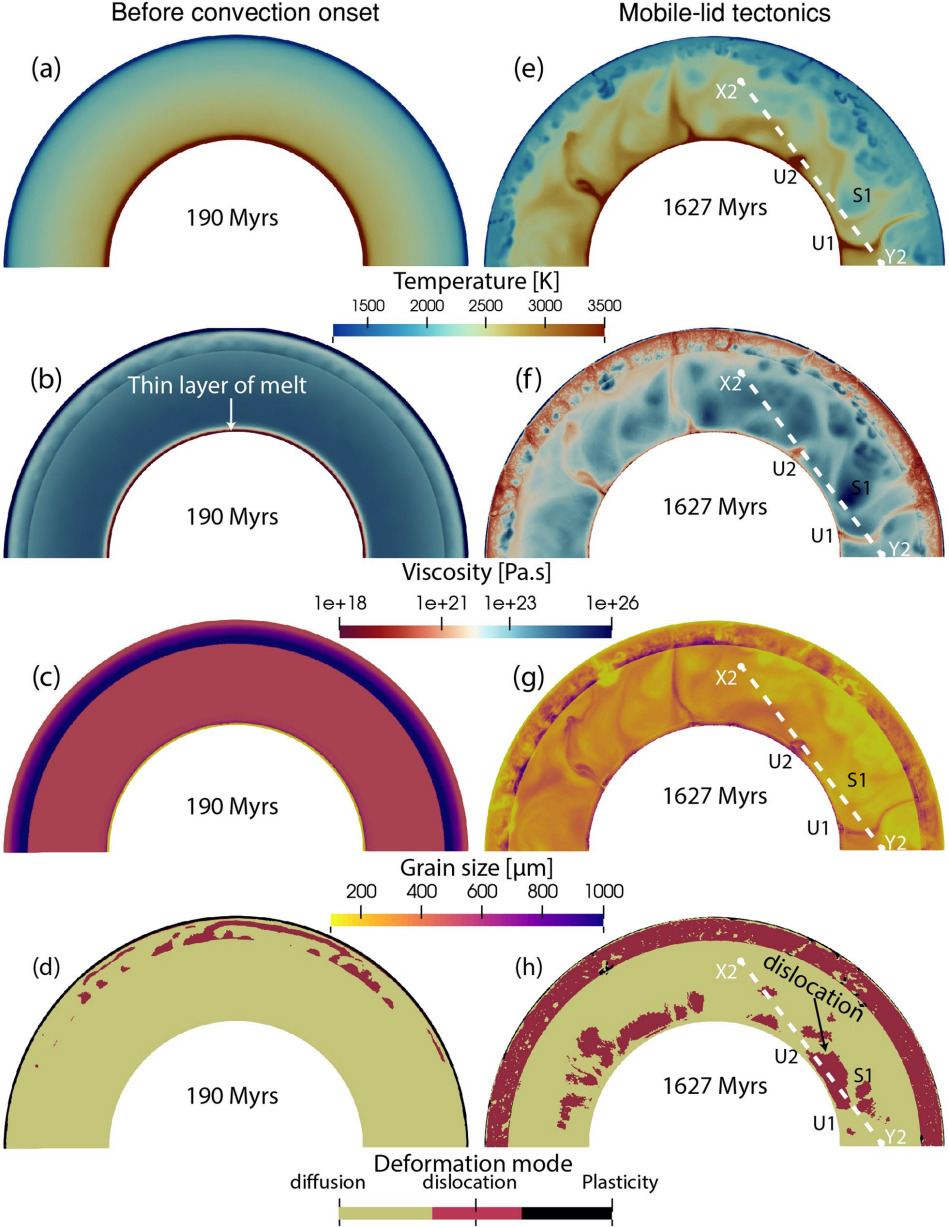
Snapshots of **a**, **e** temperature, **b**, **f** viscosity, **c**, **g** grain size and **d**, **h** deformation mode from model M2. The left panel shows snapshots at 190 Myrs and the right panel shows snapshots at 1627 Myrs. Details on profile X2-Y2, U1, U2, and S1 are discussed in Sect. 4.1

**Fig. 6:**
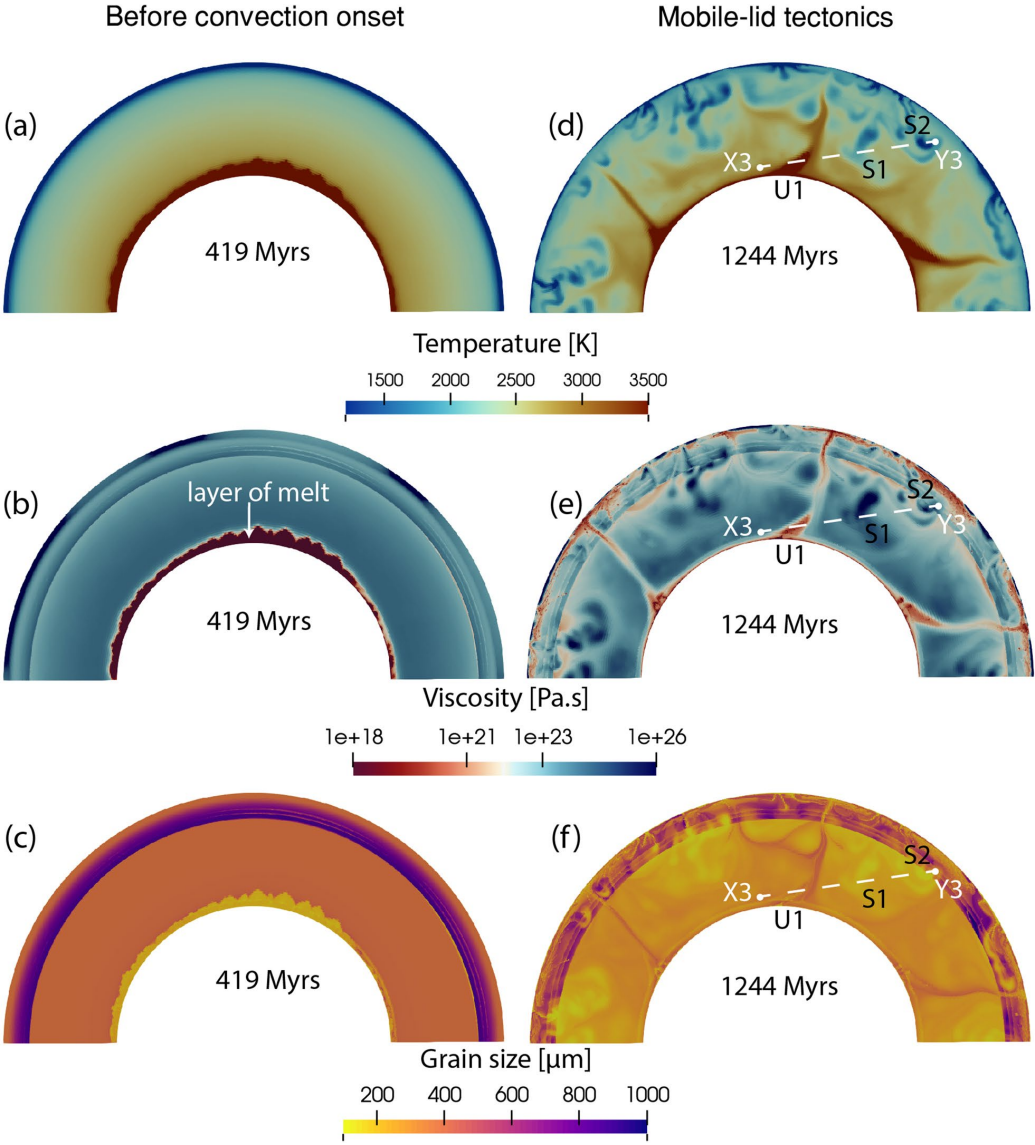
Snapshot of **a**, **d** temperature, **b**, **e** viscosity, and **c**, **f** grain size for model M3. The left panel shows snapshots at 419 Myrs and the right panel shows snapshots at 1244 Myrs. Details on profile X3-Y3, U1, S1 and S2 are discussed in Sect. 4.1

**Fig. 7:**
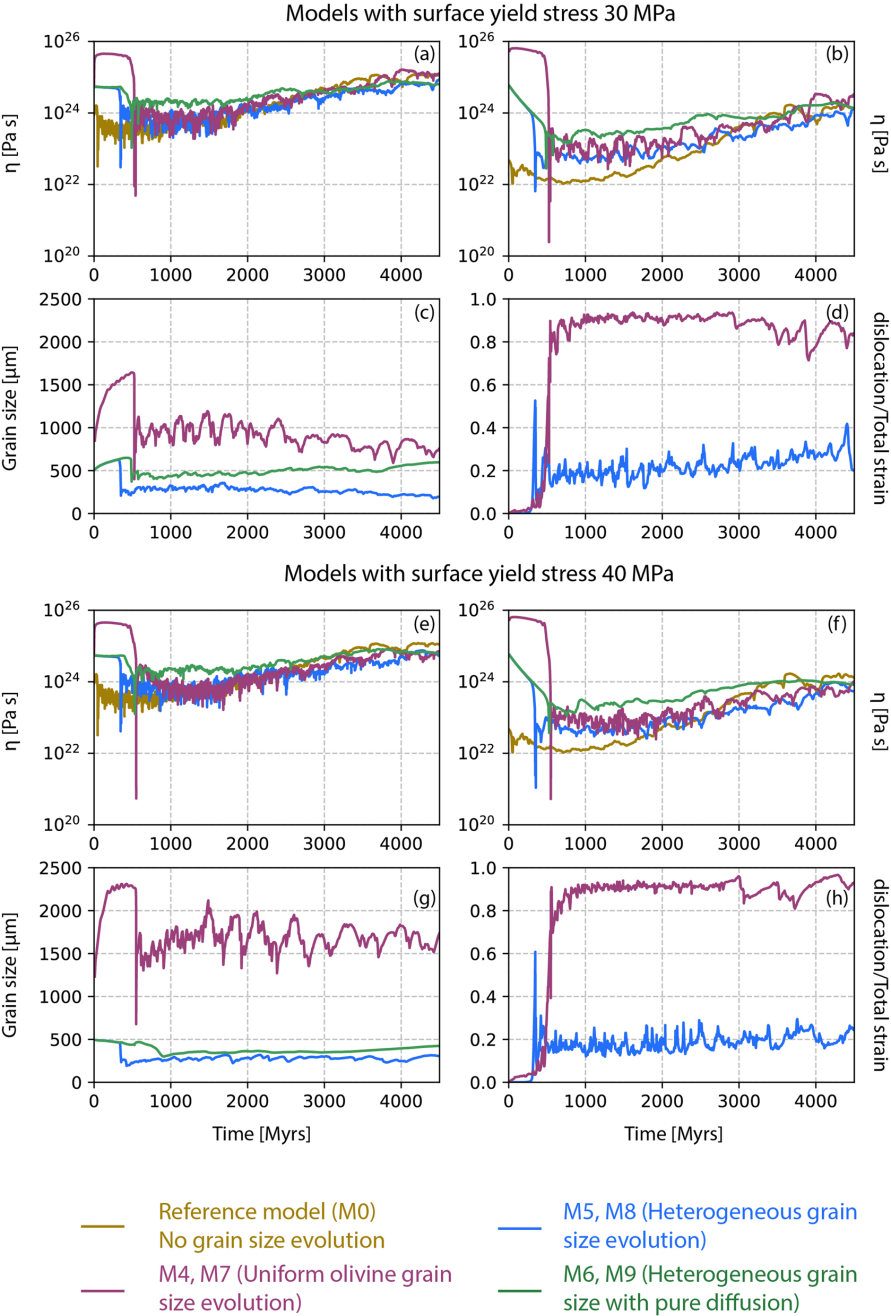
Time evolution of mantle parameters from models with different surface yield strengths. **a**–**d** Whole mantle viscosity, deep lower mantle viscosity, deep lower mantle grain size and deep lower mantle rheology from models with surface yield strength 30 MPa. **e**–**h** Same parameters from models with surface yield strength 40 MPa

**Fig. 8:**
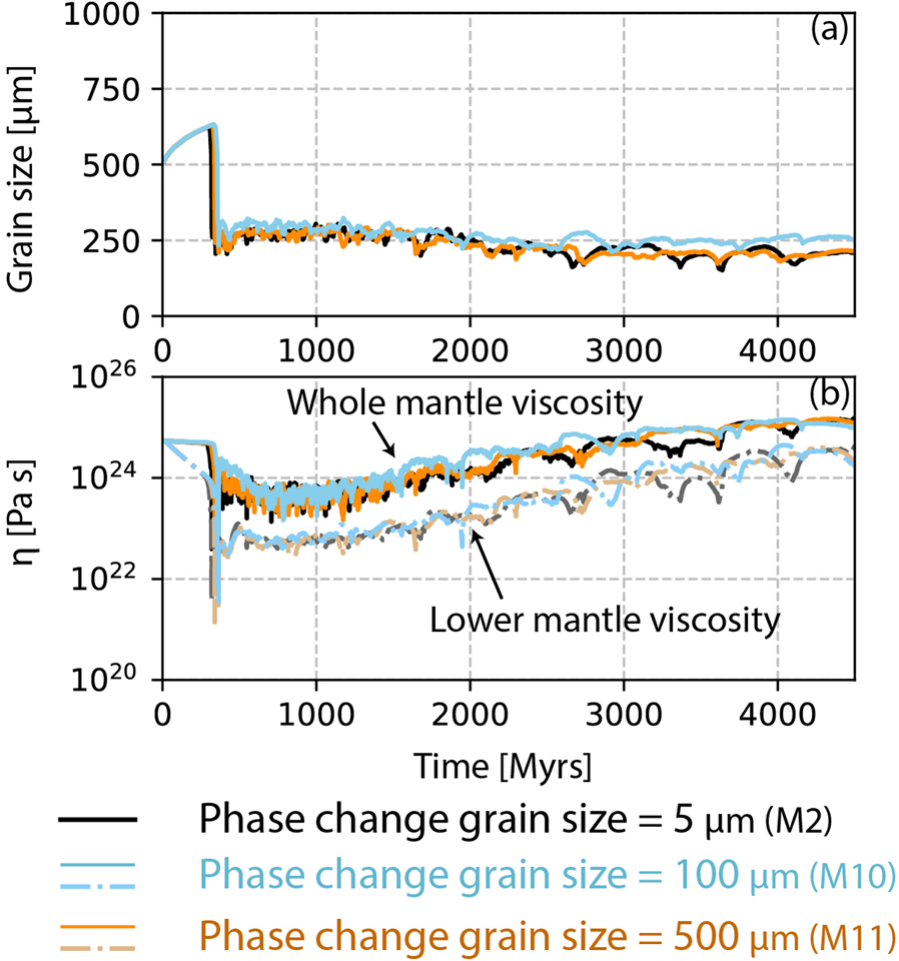
**a** Grain size distribution in the deep lower mantle, and **b** viscosity of the whole mantle (darker solid lines) and deep lower mantle (lighter dash-dotted lines) from models having different phase boundary grain size reset size

We examine different radial profiles, such as average temperature, viscosity and deformation mechanism (Fig. 2, brown lines). The temperature and viscosity profiles give values in agreement with previous literature (Fig. 2b). At the CMB, the viscosity drops well below $10^{20}$ Pa.s due to high temperature. The ratio of dislocation to the total strain rate remains close to zero, indicating that the primarily deforms in diffusion creep (Fig. 2d).

Additionally we investigate the time evolution of several parameters for both the whole mantle and the deeper lower mantle only (1000 km - CMB) (Fig. 3). With time, the average temperature of the mantle drops slowly, indicating mantle cooling over 4.5 Gyrs (Fig. 3a, e). This decrease in temperature leads to a gradual increase in viscosity (Fig. 3b). The viscosity of the whole mantle is slightly higher than that of the lower mantle because the highly viscous lithosphere is also included in the whole mantle calculation. The geometric mean of the lower mantle viscosity is at least one order of magnitude lower than the whole mantle viscosity (Fig. 3c, g). The harmonic mean of viscosity is two orders lower than the geometric mean viscosity (Fig. 3g, h). We plot both means here because it is not clear which viscosity inversion methods such as post-glacial rebound and geoid modelling are detecting. Mobility (surface velocity/average mantle velocity) remains close to 4 (Fig. 3d). The time evolution of deformation mechanism shows that diffusion creep dominates not only at 3.0 Gyrs (Fig. 2d), but consistently throughout 4.5 Gyr of evolution. The fraction of dislocation to total strain-rate remains always very small (Fig. 3i), indicating that diffusion creep is the dominant deformation mechanism in this model.

### M1: uniform grain growth in the whole mantle

In model M1 an uniform grain growth is implemented throughout the mantle using the forsterite-enstatite grain growth law (Hiraga et al. 2010). This results in a significantly larger grain size in the lower mantle, on the order of $10^4$ $\upmu$m, right from the beginning (Fig. 4c). Prior to the initiation of convection, the mantle deforms in the diffusion creep regime (Fig. 4d). The large grain size produces a mantle viscosity of $\sim 10^{26}$ Pa.s (Fig. 4b). This high viscosity restricts mobility within the mantle, leading to a temperature increase (Fig. 4a), thus the lowermost part of the mantle above the CMB begins to melt (Fig. 4b). This causes an immediate reduction in viscosity (Fig. 4b). When the thickness of the trapped melt layer grows sufficiently, it rises through the viscous mantle due to thermal buoyancy, rupturing the thick lithosphere after $\sim$ 600 million years (supplementary V3, V4). The onset of convection generates sufficient convective stress, and coupled with such a large grain size, the mantle starts deforming in dislocation creep (Fig. 4h). In this state, the net viscosity becomes independent of grain size. Consequently, the viscosity of the mantle remains primarily controlled by temperature, irrespective of the large grain size (Fig. 4e–g). Once the mobile-lid regime with slab-like downwellings is established, the large-scale dynamics of the mantle are marginally affected by grain size, and the viscosity remains unaffected.

We further quantify our findings by examining radial profiles and time-series data for various mantle properties (Figs. 2, 3, dark pink lines). The temperature profile is very similar to that of the previous reference model M0. While viscosity shows a slight increase in the lower mantle, it is not considered significant for the grain sizes of the order of $10^4$ $\upmu$m. The main reason is the self-consistent rheological partitioning between diffusion and dislocation creep. This is evident in the radial profile, where the dislocation strain-rate fraction consistently remains around 1, signifying predominant deformation via dislocation creep across the whole mantle, and making viscosity grain size-independent (Fig. 2d).

Moreover, the time evolution of average temperature, viscosity, and mobility, both in the whole mantle and specifically in the lower mantle, closely resemble the patterns observed in the reference case (Fig. 3, dark pink lines). The absence of a pronounced difference in viscosity between the models with and without grain size evolution further underscores the marginal impact of grain size on viscosity in the lower mantle, given the persistent dominance of dislocation creep.

**Fig. 9:**
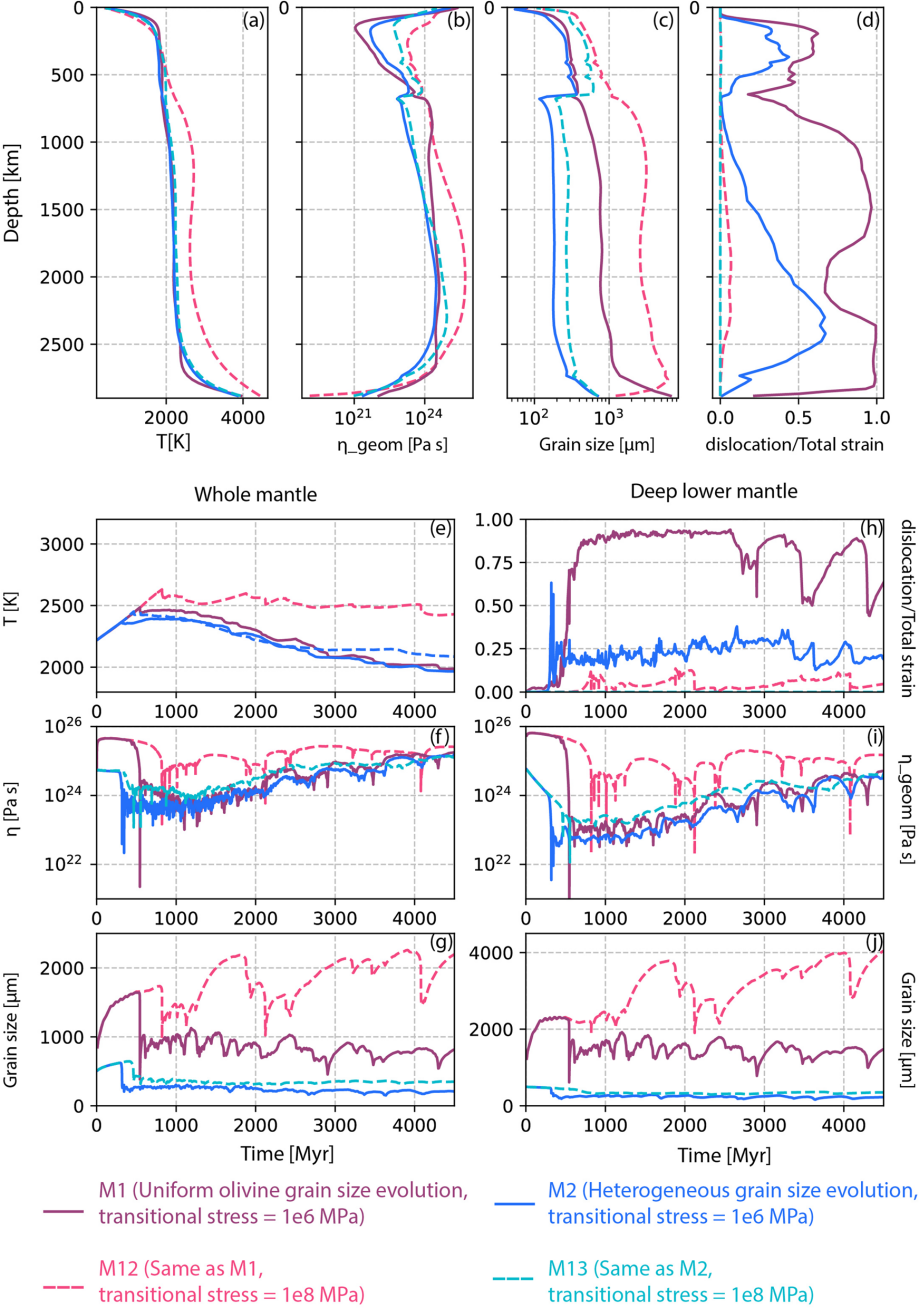
Radial profiles of **a** temperature, **b** viscosity, **c** grain size and **d** deformation mechanism at 3 Gyrs for mobile-lid tectonics. Time evolution of **e** temperature, **f**, **i** geometric mean viscosity, **g**, **j** grain size, and **h** creep mechanism. Right panel shows results for the whole mantle and left panel is for the deep lower mantle (below 1000 km depth). Four lines represent different models described in the index

**Fig. 10:**
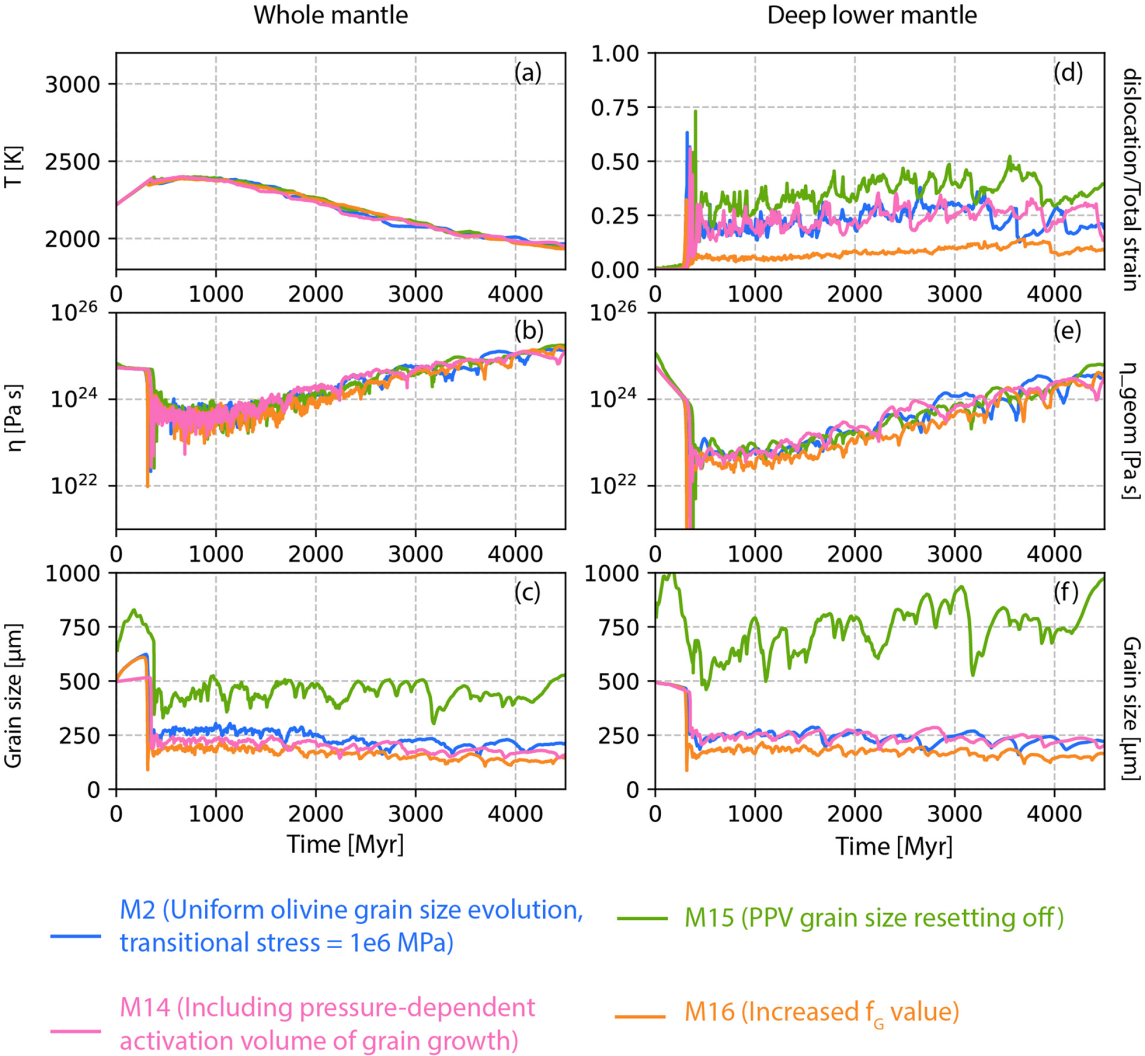
Time evolution of **a** temperature, **b**, **e** geometric mean viscosity, **c**, **f** grain size, and **d** creep mechanism. Right panel shows results for the whole mantle and left panel is for the deep lower mantle (below 1000 km depth). Four lines represent different models described in the index

**Fig. 11:**
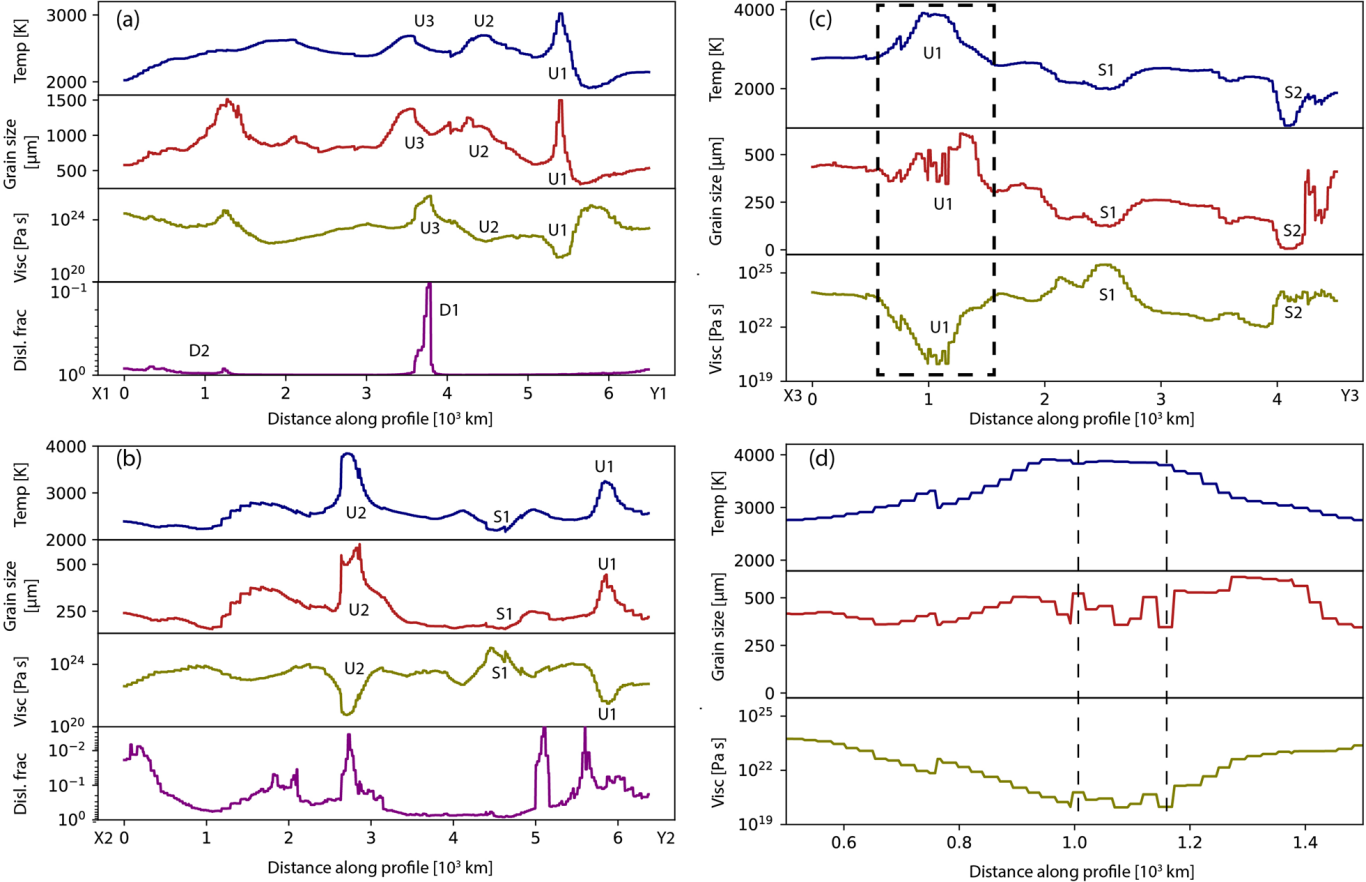
**a**, **b** Temperature, grain size, viscosity, and dislocation fraction are plotted along the X1-Y1 and X2-Y2 profiles marked in Figs. 4 and 5, respectively. **c** Temperature, grain size, and viscosity are presented along the X3-Y3 profile marked in Fig. 6. **d** A zoomed-in plot of temperature, grain size, and viscosity within the U1 region (dashed square) of subfigure c. Two vertical dashed lines are drawn to point out grain size-dependent viscosity

### M2: different grain size evolution laws in the upper and lower mantle

In model M2, we use the same setup, but introduce different grain size evolution laws in the upper and the lower mantle. In the upper mantle (0–660 km depth), grain size evolution is controlled by the forsterite-enstatite grain growth law (Hiraga et al. 2010). From 660 km depth to the CMB, grain size evolution is controlled by the recent bridgmanite-ferropericlase growth law proposed by Fei et al. (2021). Using this law, the grain growth in the lower mantle is much slower than in the upper mantle (Figs. 2c, 5c, blue line). The maximum grain size in the mantle is $\sim 10^3$ $\upmu$m (Fig. 5c, g). As in the previous model, viscosity is significantly increased before the onset of convection due to grain growth. A thin layer of melt is generated at the CMB (Fig. 5a, b), and rises within 400 Myrs, initiating mantle convection (supplementary V5, V6). Hot upwellings show the largest grain size, whereas slabs feature the smallest grain sizes (Fig. 5g). However, temperature-dependent viscosity dominates over the grain size dependency (Fig. 5e, f). After transition into the mobile-lid tectonic regime, the mantle mostly deforms in diffusion creep (Fig. 5h). Dislocation creep dominates only in regions where subducting slabs penetrate into the lower mantle.

Analysis of the radial profiles and time evolution of mantle parameters from this model (Figs. 2, 3, blue lines) shows that the temperature profile and average temperature remain very similar to those of the previous cases (Figs. 2a, 3a, e). It is important to note that the average grain size in the lower mantle is one order smaller than in model M1 (Figs. 2c, 3b, f). In model M1, the average lower mantle grain size is $\sim$ 2000 μm, while the current model M2 shows an average of $\sim$ 200 μm, even though both models started with the same initial grain size of 100 μm. Despite a significant change in grain sizes due to different growth parameters, the effect on the viscosity remains limited. Both the geometric and harmonic mean of viscosity in the lower mantle are slightly larger than in model M0, but comparable to those of model M1 (Fig. 2b, 3g, h). For model M1, where grains become very large, dislocation creep starts to dominate, while in the current model M2, grain growth is so slow that it hardly affects the mantle viscosity. Despite diffusion creep being the dominant deformation mechanism (Figs. 2d, 3i, 5h), temperature primarily controls the viscosity of the mantle. Mantle mobility in this model is comparable to those in the other two models (Fig. 3d).

### M3: pure diffusion creep

To understand the effect of composite rheology, we test a model without dislocation creep and use different grain growth parameters for the upper and lower mantle. This model behaves similarly to model M2 (Fig. 6). After a period of stagnant lid regime, widespread convection onsets after $\sim$400 Myrs (supplementary V7, V8). The transition into mobile lid tectonics with slab-like downwellings starts within the first 1.3 Gyr. Snapshots of viscosity, temperature, and grain size feature similar values compared to the previous cases. The average radial mantle temperature profile is slightly higher compared to all three previous models (Fig. 2a). We consider this to be caused by grain size-dependent rheology. As there is no dislocation creep in the upper mantle, which normally acts to decrease grain size, grains grow larger by a factor of about two compared to model M2. The average grain size in M2 is $\sim$ 300–400 μm in the upper mantle, while in model M3, this average is increased up to $\sim$ 800–900 μm (Fig. 2c, green line). This 2–3 $\times$ increase in grain size is capable of increasing the upper mantle viscosity by an order of magnitude (Fig. 2b, green line), which is clearly visible in the radial viscosity profile. Higher viscosity in the upper mantle slows down heat release from the lower mantle, causing a warmer lower mantle compared to the previous case. Under this condition, even with a slightly larger lower mantle grain size ($\sim$ 300 μm) compared to model M2 ($\sim$ 200 μm) (Figs. 2c, 3b, f, green lines), the viscosity remains similar as increased temperature counteracts the effect of the increased grain size in the current model.

### M4–M9: different surface yield strengths

The surface yield strength can influence the tectonic regime and planetary dynamics. A yield stress in the range 20–40 MPa is necessary to obtain a plate like behaviour (Lourenço et al. 2016). Thus, we have tested two additional surface yield strength values: 30 MPa (models M4–M6, Table 2) and 40 MPa (models M7–M9, Table 2). We repeated the rheology and grain size evolution law tests as performed in models M1 to M3 to determine whether the surface yield strength affects the viscosity structure. For cases with surface yield stresses of 30 and 40 MPa, the average viscosity of both the whole mantle and lower mantle remains almost the same compared to our reference model M0 (Fig. 7a, b, e, f). Due to higher surface yield strength, the lithosphere becomes more rigid, which slightly warms up the mantle. In response to this warming, models with a higher surface yield strength do have a slightly lower viscosity compared to models with 20 MPa (Fig. 3b, f). However, no significant changes due to grain size evolution are observed in our models. The average grain size in the lower mantle for higher surface yield strength models (Fig. 7c, f) remains comparable to those in models M1 and M2 (Fig. 3c, g). When forsterite-enstatite grain growth is assumed for the whole mantle, the average grain size is $\sim$ 1000 μm (Fig. 7c) for 30 MPa surface yield stress, while it is slightly larger, up to $\sim$ 2000 μm, for 40 MPa (Fig. 7g) as an effect of the mantle warming up. However, due to dislocation-dominated creep for both cases (Fig. 7d, h), the viscosity remains independent of grain size. In fact, for a slightly larger grain size, the effective viscosity in the lower mantle is reduced due to a warmer mantle. In the case of different grain growth laws in the upper and lower mantle, the grain sizes remain smaller than $\sim$ 500 μm (Fig. 7c, g). Although this grain size keeps the mantle in diffusion creep regime, such a small change in grain size does not affect mantle viscosity.

### M10–M11: effect of grain size reset at phase transitions

As additional parameter we tested the influence of the grain size reset after passing through a phase transition (Models M10–M11, Table 2). Models M1–M3 models had a very small post-transition grain size of 5 μm, close to the prediction by Solomatov and Reese (2008). We test post-transition grain sizes of 100 μm (Model M10, Table 2) and 500 μm (Model M11, Table 2) and investigate whether this increased post-transition grain size has any effect on the diffusion creep viscosity. Our results suggest that the equilibrium grain size is obtained very quickly for a particular grain growth law regardless of the phase transition grain size reset (Fig. 8a). For cases with bridgmanite-ferropericlase grain growth parameters in the lower mantle, the average grain size remains within 200–500 μm, regardless of whatever grain size reset is implemented at the phase boundary. This results in an average lower mantle viscosity comparable to previous models without visible influence of grain size (Fig. 8b).

### M12–M13: effect of transition stress

In all previous models, the reference transition stress at which diffusion creep shifts to dislocation creep was $10^6$ Pa. Given that there is no precise constraint on the value of transition stress, we have tested a few models with a transition stress of $10^8$ Pa to promote more diffusion creep in the lower mantle. Models M12 and M13 maintain all of the same input parameters as models M1 and M2, respectively, but with the higher transition stress. Model M12 adopts the uniform grain growth law of forsterite-enstatite throughout the mantle, similar to model M1. Model M13, analogous to model M2, employs different grain growth parameters in the upper and lower mantle.

We compare these new models (M12 and M13) with the earlier models featuring the lower transitional stress (M1 and M2). The radial profiles of average mantle temperature at approximately 3 Gyr reveal that the mantle warms up in model M12 compared to the others (Fig. 9a, dashed pink line). However, despite the higher temperature, the corresponding viscosity profiles indicate that M12 displays an increased viscosity in the lower mantle (Fig. 9b). This positive correlation between temperature and viscosity can be attributed to the larger grain size in model M12 (Fig. 9c). With the increased transition stress, the lower mantle deforms mostly in diffusion creep (Fig. 9d) and the viscosity remains strongly grain size dependent. It is notable that model M13 does not exhibit any significant differences in lower mantle viscosity and temperature (Fig. 9a, b, dashed cyan line), even with the elevated transitional stress.

The time-series analysis of these models identifies a similar pattern. Model M12 displays higher temperature alongside increased viscosity due to the larger grain size throughout the mantle (Fig. 9e, f, i). Diffusion creep dominates in models M2, M12, and M13, while dislocation creep is only prevalent in model M1, as discussed previously (Figs. 3i, 9h). The absence of dislocation leads to even larger grain growth in M12 as compared to M1 (Fig. 9g, j). Throughout the mantle, average grain size remains between 1000–2000 μm, while in the lower mantle, the average grain sizes reach up to 4000 μm. The time series analysis also confirms that increasing the transition stress to $10^8$ MPa does not significantly impact model M13, and shows analogous behavior to M2 (Fig. 9f, i). This is because in M2, the lower mantle was primarily undergoing diffusion creep, except in a few regions with downwellings. Increasing transitional stress in model M13 produces results very similar to M3, where pure diffusion creep has been imposed everywhere.

### M14–M16: additional parameter tests

We also tested three additional models, each incorporating a modification related to the grain growth law. One important parameter could be the effect of pressure on grain growth equation (Okamoto and Hiraga 2022, 2024; Zhang and Karato 2021). In model M14, we modify the grain growth Eq. (8) as $\text{G} = \text{G}_0 \text{exp} \left( \frac{-\text{E}_{\text{G}} + \text{PV}_\text{G}}{\text{RT}} \right)$, where $\text{E}_{\text{G}}$ is activation energy, $\mathrm {V_{G}}$ is activation volume. $\text{V}_{\text{G}}$ has a pressure dependence following the Eq. (4). Previous studies proposed a common diffusion mechanism both for grain growth and creep (Okamoto and Hiraga 2022, 2024). For this purpose, we employ the values for $\text{E}_{\text{G}}$, $\text{V}_{\text{G}}$ and $\text{P}_{\text{decay}}$ as used for diffusion creep for the lower mantle below 660 km (Table 1). The results show that incorporating these parameters has almost no effect compared to the parameters used previously for model M2. A small change in average grain size of the whole mantle is observed, but for the lower mantle the average grain size remains the same (Fig. 10c, f). The average temperature, viscosity, and creep mechanism remain almost the same for model M14 (light pink lines in Fig. 10) when compared with model M2 (blue lines in Fig. 10).

In model M15, the resetting of grain size at the post-perovskite boundary is disabled. This results in an increased average grain size across the whole mantle as well as in the lower mantle (Fig. 10c, f; light green lines), while the average viscosity remains the same when compared to other models.

To enhance grain damage, we increase in model M16 the $f_{bot}$ of grain size evolution parameter (Eq. 9) to $10^{-3}$ (Table 2), keeping all other parameters the same as in model M2. Due to amplified grain damage, the average grain size is slightly reduced in model M16 (Fig. 10c, f; orange lines) when compared to model M2 (Fig. 10, blue lines). However, if the lower mantle average grain size is controlled by the bridgmanite-ferropericlase growth parameters, the mantle viscosity remains primarily controlled by temperature.

## Discussion

### Temperature-dependent viscosity is stronger than grain size-dependent viscosity

Over the past two decades, various models were proposed featuring the influence of grain size-dependent viscosity (Dannberg et al. 2017; Hall and Parmentier 2003; Korenaga 2005; Rozel et al. 2011; Rozel 2012; Schierjott et al. 2020; Solomatov 1996; Solomatov et al. 2002; Solomatov and Reese 2008). While it seems plausible that grain size could significantly influence mantle viscosity (Karato 1984; Fei et al. 2023), most of our models do not show a significant variation in viscosity due to grain size evolution (Figs. 2, 3). Some difference occurs in models M3, M6, M9 and M12. In models M3, M6 and M9 dislocation creep is absent and in the model M12 the transition stress is increased to $10^8$ Pa. All other models, regardless of surface yield stress, grain size reset, different grain damage parameters, and transition stress, show no difference in the average lower mantle viscosity. There are two main reasons for these results. First, the slow grain growth of the lower mantle mineral assemblage remains subdued due to the temperature dependence of viscosity. Secondly, given the limited data on lower mantle grain growth (Fei et al. 2021; Yamazaki et al. 1996), we tested a hypothetical scenario using olivine grain growth parameters throughout the whole mantle to artificially increase grain size in the lower mantle. While these models are clearly unrealistic, they offer insights into the creep mechanisms when large grains are present. The numerical models indicate that in the presence of such large grains, dislocation creep dominates, rendering viscosity independent of grain size.

The two-phase mixture of lower mantle minerals, including bridgmanite-ferropericlase, grows extremely slowly in the lower mantle (Fei et al. 2021). The Zener pinning effect of ferropericlase significantly suppresses the growth of the bridgmanite-ferropericlase assemblage (Fei et al. 2023). When grain size evolution parameters are controlled by bridgmanite-ferropericlase grain growth, the average grain size remains less than 1000 μm. This estimate is consistent with the observations of Okamoto and Hiraga (2022), who also estimated the maximum grain size of the lower mantle to be close to 900 μm. Such a small average grain size is unable to overcome the effects of temperature-dependent viscosity. Only in model M13 do we find a significant effect of grain size dominating over temperature-dependent viscosity, but achieving such a large grain size in the present-day Earth is unrealistic. The Zener pinning of bridgmanite-ferropericlase restricts grain size to much smaller values, as observed in more realistic models with bridgmanite-ferropericlase grain growth parameters.

In models M3, M6 and M9, we have turned off dislocation creep and hence diffusion creep dominates in the upper mantle. These models show larger grain size in the upper mantle caused by faster forsterite-enstatite grain growth that elevates the viscosity of the upper mantle by roughly an order of magnitude (Fig. 2b, c). However, the lower mantle remains unaffected due to the slower grain growth of bridgmanite-ferropericlase.

To quantitatively investigate the effect of grain size in the lower mantle, we compare temperature, grain size, viscosity, and dislocation fraction along three profiles taken from models M1–M3 (Fig. 11). Along the X1-Y1 profile in model M1, we identify three upwellings as U1, U2, and U3 (Fig. 4), corresponding to three temperature peaks (Fig. 11a). The grain size evolution follows the temperature curve, with regions U1 and U2 showing a drop in viscosity despite larger grain size. However, the U3 region shows a viscosity increase, indicating the effect of grain size evolution. Analysis of creep fraction reveals that diffusion creep is the dominant creep in this region (D1), making viscosity sensitive to grain size. On the left side of the profile, where the diffusion creep fraction slightly increases (D2), viscosity tends to follow the grain size curve as the grains are sufficiently large in this model. However, as most of the lower mantle in this model remains in dislocation creep, viscosity remains insensitive to grain size even if it is large.

For model M2, the insignificant effect of grain size is more clearly visible. Along the X2-Y2 swath (Fig. 5), we consider two hot upwelling regions (U1, U2) and a cold subduction region (S1). The temperature curve clearly shows these three regions (Fig. 11b), and the grain size curve correlates well with the temperature curve. The viscosity curve inversely follows the temperature curve without showing any grain size effect, even though the primary deformation mechanism mostly remains in diffusion creep.

In model M3, we observe a similar trend along the X3-Y3 profile of temperature, grain size, and viscosity (Fig. 11c). In the upwelling region (U1), the temperature is high, corresponding to a large grain size and lower viscosity. The two subduction regions (S1, S2) show both a lower temperature, smaller grain size, and higher viscosity. However, small scale crenulations are observed in the grain size and viscosity curves. Zooming into the U1 region (dashed square) reveals that the long-wavelength viscosity curve follows the temperature, while the small-scale fluctuations do follow the grain size variation (follow two dashed lines in Fig. 11d). However, these second-order effects are too small to change the average mantle viscosity.

Our results agree with the previous study by Schierjott et al. (2020), who did not find a significant difference in viscosity between thermochemical piles and their surroundings, with the piles having a larger grain size. Dannberg et al. (2017) found some significant changes in the viscosity of plumes and slabs due to grain size variations. However, in their phase-dependent rheology, bridgmanite was assumed to be deforming purely by diffusion creep. We implemented a self-consistent rheology that allowed the lower mantle to deform in dislocation creep when required. Unlike Schierjott et al. (2020), we did not enforce diffusion creep in the lower mantle using an additional diffusion creep efficiency factor, providing a more realistic approximation. While calculating the viscosity jump in the lower mantle, Fei et al. (2023) assumed that the lower mantle deformation is governed by pure diffusion creep, which may not be a very realistic situation for the real Earth.

The recent grain growth data for bridgmanite-ferropericlase show an extremely slow growth rate (Fei et al. 2021) compared to forsterite-enstatite growth in the upper mantle (Hiraga et al. 2010), that does not allow the equilibrium grain size in the lower mantle to exceed 200–300 μm. Such slow grain growth actually allows the mantle to deform mostly in diffusion creep, but the effect of grain size is often overshadowed by the effect of temperature in the lower mantle. Fei et al. (2023) suggested that pure bridgmanite may display a much faster grain growth than the bridgmanite-ferropericlase assemblage. In our models we observed that a larger grain size may cause deformation in dislocation creep, making the viscosity independent of grain size.

### Anisotropy and dislocation creep within the lower mantle

Our understanding of lower mantle viscosity is limited, and measurements from mineral physics experiments (Karato 2008) feature uncertainties due to the short time-scale of such experiments. Apart from mineral physics experiments, lower-mantle viscosity has been estimated by various geophysical inversions of geoid, post-glacial rebound, dynamic topography, slab sinking speed, etc. (e.g Čížková et al. 2012; Forte and Mitrovica 1996; Panasyuk and Hager 2000; Steinberger and Calderwood 2006; Lau et al. 2016). These studies suggested that the lower mantle viscosity ranges from $10^{22}$ to $10^{24}$ Pa.s. Our models display a geometric mean lower-mantle viscosity of $\sim 10^{23}$ Pa.s in the shallower part (up to 1000 km), reaching $\sim 10^{24}$ Pa.s with increasing depth (Fig. 2b). Below 2500 km depth, the viscosity starts to drop and reduces to $10^{20}$ Pa.s. Due to mantle cooling in our models, viscosity increases with time. The geometric mean of lower mantle viscosity is estimated to be close to $10^{24}$ Pa.s, whereas the harmonic mean is $\sim 10^{22}$ Pa.s (Fig. 3g, h). It is not clear which of these means most closely corresponds to what the above observational constraints are detecting.

While earlier studies suggested that diffusion creep may be the dominant deformation mechanism (Karato et al. 1995; McNamara et al. 2002), there is evidence for dislocation creep in lower mantle mineral assemblages. Yamazaki and Karato (2002) experimentally observed the development of a fabric in the periclase system ((Mg,Fe)O) during shearing. Cordier et al. (2004) observed the development of crystal-preferred orientation (CPO) in deformation experiments on coarse polycrystals of bridgmanite at 25 GPa and 1400 K. In another high-pressure experiment, Miyagi and Wenk (2016) showed that the enstatite to bridgmanite transformation can develop a slip (001) system, which could be the dominant source of anisotropy in the lower mantle. Numerical models by McNamara et al. (2002) suggested that most slabs deform by dislocation creep in the lower mantle. The recent global seismic anisotropy model SGLOBE-rani (Ferreira et al. 2019) indicated that the topmost part of the lower mantle is anisotropic, particularly underneath subduction zones. This anisotropy could be due to lattice-preferred orientation caused by dislocation creep. Therefore, dislocation creep is expected to be an important deformation mechanism in some regions of the lower mantle, especially in the downwelling regions.

Another view on the deformation mechanism in the lower mantle is that it deforms through pure climb dislocation creep (Boioli et al. 2017) rather than climb-assisted glide, which is the classical dislocation-creep mechanism (Karato 2008). The macroscopic flow law of pure climb creep is similar to that of classical dislocation creep (Reali et al. 2019) and the transition grain-size between diffusion creep and dislocation creep may be in a similar range (comparing the predictions of Reali et al. (2019) and Tsujino et al. (2022)), so the assumed dislocation-creep mechanism does not affect our presented results. A major difference, however, is that pure climb does not cause CPO and associated anisotropy (Boioli et al. 2017). Reali et al. (2019) predicted that the lower mantle is undergoing pure climb dislocation creep if the lower mantle grain size is larger than about $10^2$–$10^3$ $\upmu$m, in which case the rheology is grain size insensitive. This is in line with our results, where for larger grain sizes, dislocation creep dominates. In this scenario, the effect of grain size on lower mantle viscosity is negligible. However, further investigation will be required to establish the ubiquitous occurrence of this creep mechanism in the lower mantle.

M2 and M13 are the most optimized and realistic models in our study, that incorporate recent grain size evolution data along with a composite rheology. Model M2 shows dislocation-dominated regions only in the downwelling regions of the lower mantle, while most other regions experience diffusion creep. This model qualitatively agrees with the seismic model of Ferreira et al. (2019). In contrast, the reference model M0, which does not include grain size evolution, shows a completely diffusion-dominated deformation throughout the lower mantle.

Model M1 assumes the grain growth law for forsterite-enstatite to govern in the lower mantle, which is clearly unrealistic, and suggests that the lower mantle is completely dominated by dislocation creep. This dislocation creep dominance is a consequence of the unusually large grain size within the lower mantle due to the use of the olivine grain growth law by Hiraga et al. (2010). The olivine growth law produces an order of magnitude larger grain size (up to 15000 μm) than predicted by the bridgmanite-periclase grain growth law of Fei et al. (2021) (up to 1000 μm) (Fig. 2c). Obtaining dislocation creep for large grain sizes is not unrealistic (Solomatov and Reese 2008). Previous experimental studies on bridgmanite have already shown that at the top of the lower mantle (25 GPa, 1900 K), grain sizes of the order of $10^{4}$ $\upmu$m likely favour dislocation creep even at a very low stress of $10^4$ Pa (Tsujino et al. 2022). Therefore, the dislocation-dominated lower mantle in model M1 may be unrealistic for the real Earth scenario, while our implementation of self-consistent rheology partitioning is in agreement with experimental, seismic, and previous geodynamic models.

### Relevance of grain size in early Earth dynamics

Even if we find almost no effect of grain size on present-day Earth lower mantle viscosity, it may still have been relevant in early Earth dynamics. When comparing lower mantle scenarios, models with grain size evolution exhibit a slightly higher viscosity before the onset of convection (Figs. 3f, 7b, f). In models like M1 and M12, where we have enforced a large grain size in the lower mantle, might also be relevant for the early Earth. Some studies suggest that bridgmanite crystals could form as a dominant phase from the basal magma ocean (Xie et al. 2020). In the absence of a secondary phase (e.g., ferroperilcase) Zener pinning effect would be less important and bridgmanite grains can grow significantly larger (Fei et al. 2023), subsequently increasing the viscosity of the lower mantle in the early Earth. A high-viscosity lower mantle could delay the lid-breaking event and the onset of global convection in early Earth. The larger the grain size in the early Earth, the more time it takes to initiate convection and the lid-breaking event. For example, models M1 and M12 show lid-breaking occurring at $\sim$ 600–800 Myr, whereas models with smaller grain sizes feature lid-breaking events occurring much earlier, within 200–300 Myr.

## Conclusions and outlook

Our mantle convection models, integrating the latest grain growth parameters and composite rheology, consistently suggest that temperature-dependent viscosity dominates over grain size-dependent viscosity in present-day Earth. The most recent dataset for bridgmanite-ferropericlase produces smaller grain sizes in the lower mantle. With these small grain sizes, mantle viscosity is minimally affected, as temperature remains the dominant factor governing viscosity. If we force large grain size in the lower mantle to test the end member scenario, the mantle starts deforming in dislocation creep making viscosity essentially independent of grain size. This aligns with experimental studies, which indicate that dislocation creep is associated with larger grain sizes. There is scope for further experimental improvements of both the grain growth as well as the grain damage parameters, however these are likely to have only marginal effects on average mantle viscosity. In a pure diffusion creep model, there is a weak second order effect of grain size, while the temperature effect on viscosity remains dominant. Previous ideas about slowing down mantle mixing due to large grain size (Solomatov and Reese 2008; Foley and Rizo 2017) may not be as significant when dislocation creep is taken into account. Our first order results may infer that, if single phase crystals were forming in the basal magma ocean, the mantle viscosity on early Earth could be significantly higher. Such a highly viscous mantle could delay the lid-breaking event on early Earth. Once convection starts and secondary phases appear in the lower mantle, the grains can not grow large and the temperature effect starts to dominate viscosity. The secondary phase, i.e., ferropericlase tends to reduce the effective viscosity, especially when it forms an interconnected ferropericlase network (Thielmann et al. 2020) under large stress conditions. Gülcher et al. (2022) showed that strain weakening could produce narrow and cold plumes in the lower mantle. It might be useful to develop studies that combine strain weakening and grain size evolution in the lower mantle to investigate large-scale dynamics.

Finally, it might also be interesting to explore small-scale geodynamic problems in more detail, particularly in lithospheric shear zones where grain size can decrease rapidly. This issue could potentially impact the viscosity of sheared regions, as small grain sizes will promote a diffusion creep regime where viscosity is strongly grain size-dependent. Grain size-dependent viscosity could also be relevant for understanding shallow mantle processes, such as thermal runaway during earthquake processes (Thielmann et al. 2015; Spang et al. 2024) or craton stabilization due to grain growth (Lee and Chin 2023). Such scenarios deserve further study to understand the effect of grain size in more detail.

## Additional file

Supplementary file 1

Supplementary file 2

Supplementary file 3

Supplementary file 4

Supplementary file 5

Supplementary file 6

Supplementary file 7

Supplementary file 8

Supplementary file 9

## Data Availability

All required data are given in tables along with the main manuscript.
