# Supplementary material for: Effect of bridgmanite-ferropericlase grain size evolution on Earth’s average mantle viscosity: implications for mantle convection in early and present-day Earth
Source: Prog Earth Planet Sci. 2024 Dec 4;11(1):64. doi: 10.1186/s40645-024-00658-3 (PMC11615032; doi:10.1186/s40645-024-00658-3)
Supplement: Supplementary file 1 [file 40645_2024_658_MOESM1_ESM.docx]

V1 and V2: Animation of temperature and viscosity from model M0

V3 and V4: Animation of temperature and viscosity from model M1

V5 and V6: Animation of temperature and viscosity from model M2

V7 and V8: Animation of temperature and viscosity from model M3
